# Serotonin Transporter Genetic Variation and Antidepressant Response and Tolerability: A Systematic Review and Meta-Analysis

**DOI:** 10.3390/jpm11121334

**Published:** 2021-12-09

**Authors:** Kiera Stein, Abdullah Al Maruf, Daniel J. Müller, Jeffrey R. Bishop, Chad A. Bousman

**Affiliations:** 1 Department of Medical Genetics, University of Calgary, Calgary, AB T2N 4N1, Canada; kiera.stein@ucalgary.ca; 2 College of Pharmacy, Rady Faculty of Health Sciences, University of Manitoba, Winnipeg, MB R3E 0T5, Canada; abdullah.maruf@umanitoba.ca; 3 The Mathison Centre for Mental Health Research & Education, Hotchkiss Brain Institute, Cumming School of Medicine, University of Calgary, Calgary, AB T2N 4N1, Canada; 4 Department of Psychiatry, University of Calgary, Calgary, AB T2N 4N1, Canada; 5 Pharmacogenetics Research Clinic, Campbell Family Mental Health Research Institute, Centre for Addiction and Mental Health, Toronto, ON M6J 1H4, Canada; daniel.mueller@camh.ca; 6 Department of Psychiatry, University of Toronto, Toronto, ON M5S 1A1, Canada; 7 Department of Psychiatry, Psychosomatics and Psychotherapy, University of Würzburg, 97078 Würzburg, Germany; 8 Department of Experimental and Clinical Pharmacology, University of Minnesota, Minneapolis, MN 55455, USA; jrbishop@umn.edu; 9 Alberta Children’s Hospital Research Institute, University of Calgary, Calgary, AB T2N 4N1, Canada

**Keywords:** 5-HTTLPR, *SLC6A4*, genotype, pharmacogenetics, antidepressant, efficacy, tolerability

## Abstract

Antidepressants are used to treat several psychiatric disorders; however, a large proportion of patients do not respond to their first antidepressant therapy and often experience adverse drug reactions (ADR). A common insertion–deletion polymorphism in the promoter region (5-HTTLPR) of the serotonin transporter (*SLC6A4*) gene has been frequently investigated for its association with antidepressant outcomes. Here, we performed a systematic review and meta-analysis to assess 5-HTTLPR associations with antidepressants: (1) response in psychiatric disorders other than major depressive disorder (MDD) and (2) tolerability across all psychiatric disorders. Literature searches were performed up to January 2021, yielding 82 studies that met inclusion criteria, and 16 of these studies were included in the meta-analyses. Carriers of the 5-HTTLPR LL or LS genotypes were more likely to respond to antidepressant therapy, compared to the SS carriers in the total and European ancestry-only study populations. Long (L) allele carriers taking selective serotonin reuptake inhibitors (SSRIs) reported fewer ADRs relative to short/short (SS) carriers. European L carriers taking SSRIs had lower ADR rates than S carriers. These results suggest the 5-HTTLPR polymorphism may serve as a marker for antidepressant outcomes in psychiatric disorders and may be particularly relevant to SSRI treatment among individuals of European descent.

## 1. Introduction

Antidepressant medications are commonly used to treat several mood and anxiety disorders such as major depressive disorder (MDD), obsessive compulsive disorder (OCD), generalized anxiety disorder (GAD), and social anxiety disorder. However, roughly 40% of patients taking antidepressants experience at least one side effect [1]. Furthermore, only 50–60% of patients with uncomplicated MDD respond to any single antidepressant [2]. Antidepressant outcomes depend on a number of factors, including genetic variation, which contributes to about 42–50% of antidepressant response rates [3]. Therefore, decision support tools have been developed to assist physicians’ prescribing decisions based on an individual’s genotype [4].

In psychiatry, these pharmacogenetic-guided “decision support tools” have primarily included genes involved in antidepressant pharmacokinetics (e.g., cytochrome P450 genes). This is due to their established relationships with drug exposure, implications for dosing, and also the availability of dosing guidelines developed by expert groups such as the Clinical Pharmacogenetics Implementation Consortium (CPIC) and the Dutch Pharmacogenetics Working Group (DPWG) [5,6]. However, pharmacodynamic genes such as the serotonin transporter (*SLC6A4*) also appear on many commercially available pharmacogenetic tests [4], despite the absence of dosing or drug selection guidelines [7]. Some studies suggest that a 43-base pair insertion (long allele) or deletion (short allele) polymorphism (rs4795541) in the promoter region of the *SLC6A4* gene, known as 5-HTTLPR, is associated with the response to selective-serotonin reuptake inhibitors (SSRIs) [8,9,10,11,12]. Specifically, these studies have reported that patients with the long/long (LL) or long/short (LS) genotypes have a better response to SSRIs compared to patients with the short/short (SS) genotype. This association is supported by in vitro data showing that the L allele is associated with greater serotonin transporter expression relative to the S allele [13], as well as in vivo data that have demonstrated SSRIs directly bind to the serotonin transporter protein, inhibiting the recycling of serotonin [14]. However, some studies have found that patients with the SS or SL genotype have improved treatment outcomes when compared to patients with the LL genotype [15,16], while others have reported no association with treatment outcomes [17,18,19]. These mixed results have, in part, been attributed to other polymorphisms in the promoter of the *SLC6A4* gene, such as rs25531A > G. This variant is often used to sub-divide the L allele into L_A_ and L_G_. When sub-divided, some studies have shown that the L_A_ confers greater expression of the serotonin transporter, while the L_G_ has similar expression to the S allele [20,21]. However, contrary findings have been published [22], and consensus on whether it is clinically useful to genotype the rs25531 variant in combination with the 5-HTTLPR polymorphisms has not been reached.

To address these conflicting findings, Ren and colleagues [23] recently published a meta-analysis that showed the 5-HTTLPR L allele was associated with better antidepressant response in patients diagnosed with MDD, particularly those of European ancestry. However, it is unclear whether 5-HTTLPR genetic variation is associated with antidepressant response among individuals with psychiatric disorders other than MDD, nor is it clear whether this polymorphism is associated with antidepressant-induced adverse drug reactions (ADRs) across psychiatric disorders.

To address this gap in the knowledge base and inform future prescribing guidelines, we performed a systematic review and meta-analysis that examined 5-HTTLPR associations with: (1) antidepressant response among individuals with psychiatric disorders other than MDD and (2) antidepressant tolerability among individuals with any psychiatric disorder.

## 2. Materials and Methods

### 2.1. Search Strategy and Selection Criteria

The systematic review was registered with PROSPERO (registration number: CRD42020170164) and followed the 2020 PRISMA (Preferred Reporting Items for Systematic Reviews and Meta-Analyses) reporting recommendations [24]. Two reviewers (K.S. and A.M.) independently searched PubMed and Google Scholar up to January 2021. The search strategy was: (((SLC6A4 OR serotonin transporter OR 5-HTT)) AND (antidepressant OR tricyclic OR SSRI OR SNRI OR MAOI)) AND (pharmacogenetic OR allele OR genotype OR variant). Bibliographies of all research articles were hand-searched for additional references not identified in our primary searches. Both reviewers independently assessed all articles identified by the search strategy for eligibility. Articles for which a consensus between the two reviewers was not obtained were evaluated by a third reviewer (C.B.).

Studies published in English were selected for data extraction and analysis based on the following inclusion criteria: (1) examined individuals treated with antidepressant medication, (2) genotyping of the 5-HTTLPR polymorphism was conducted and results were reported, and (3) performed assessment of symptom severity, response, remission, and/or ADRs among individuals with a psychiatric disorder. During the inclusion/exclusion process, Ren and colleagues [23] published a meta-analysis that assessed the association between the 5-HTTLPR polymorphism and antidepressant treatment response in MDD. To avoid redundant results in our response analyses, we excluded studies that only reported associations between 5-HTTLPR and antidepressant response in MDD patients. However, MDD studies that contained sufficient ADR data were retained for our tolerability analyses.

Two independent reviewers (K.S. and A.M.) used a custom data extraction template to summarize the selected articles. Extraction information included author names, year, study design, sample size, recruitment site, type of antidepressant, other medications used, patient characteristics (i.e., age, sex, ethnic mix, comorbidities), diagnosis, phenotype assessed, and phenotypic measures. When information was missing or incomplete for an eligible study, a request for additional information was made to the corresponding author of the eligible study.

### 2.2. Quality Review

An assessment of study quality was conducted independently by the two reviewers (K.S. and A.A.M.), which we adapted from a checklist developed by Jorgensen and Williamson [25]. For each of the questions included in the quality assessment tool, a “Yes” was recorded if the study definitively and affirmatively addressed the question; otherwise, a “No” for the question was recorded. The number of “yes” responses was summed to derive a quality score (range 0–26) for each included study, where higher scores suggest a higher quality study.

### 2.3. Data Analysis

Data were analyzed using the *Major* package in Jamovi version 1.2.27.0 [26] and Cochrane Review Manager, RevMan 5. The odds ratio (OR) was used as the effect size estimator and was calculated by contrasting the counts of antidepressant response (defined by each included study as exceeding a threshold percent decrease in symptom severity) or ADRs (defined as the presence of one or more assessed ADRs) within 5-HTTLPR genotype groups. Three genetic models were examined, which included the allelic model (L vs. S), dominant model (LL/LS vs. SS), and the homozygous model (LL vs. SS). The pooled ORs were calculated using a random-effects model for dichotomous data, which was the Mantel–Haenszel method. Heterogeneity in effect sizes between studies was tested using the chi-square statistic (with *p* < 0.10 indicating significant heterogeneity), and its magnitude was quantified using the I-squared statistic, which is an index that describes the proportion of the total variation in the study effect size estimates that is due to heterogeneity and is independent of the number of studies included in the meta-analysis and the metric of the effect size. Publication bias was evaluated using funnel plots and Egger’s regression test [27] for funnel plot asymmetry. Following the recommendations of Dalton et al. [28], a test for funnel plot asymmetry was only conducted if the number of studies was 10 or greater. These practices are in line with the guidelines for conducting a meta-analyses outlined in the Cochrane Handbook [29].

Subgroup analysis with respect to ancestry was conducted, as well as clinical diagnosis, presence of the *SLC6A4* rs25531A > G genotype, and antidepressant class (SSRI vs. non-SSRI) when three or more studies were available. Moderator analyses for participant sex, age, and ancestry (European vs. non-European) were conducted using mixed effects meta-analyses with the DerSimonian–Laird random-effects model.

## 3. Results

**Figure 1:**
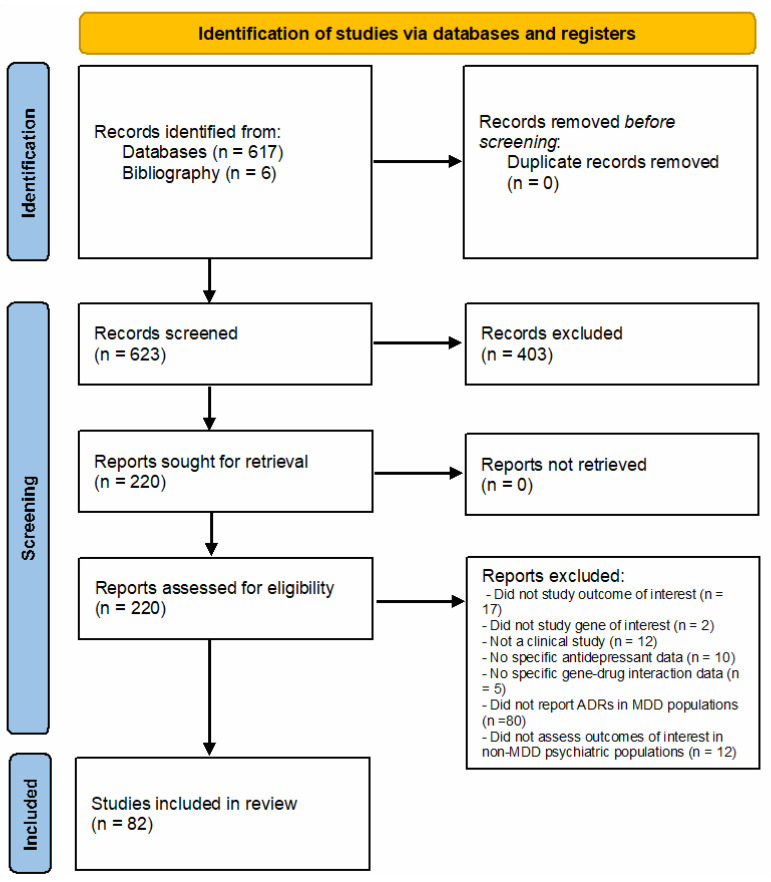
PRISMA flow chart detailing the article selection process.

Our systematic search yielded a total of 623 studies. A summary of the article selection process can be found in Figure 1. After reviewing titles and abstracts, 403 studies were excluded, because they did not meet the study eligibility criteria. After the full text screening of the remaining 220 articles, 46 articles were excluded. After the Ren et al. [23] meta-analysis had been published, 88 articles were further excluded in order to prevent redundant findings. Summary characteristics of the remaining 82 articles is presented in Table 1. A detailed summary of each article can be found in Supplementary Table S1. Most of the studies were of fair-to-moderate quality, and none of the studies met all the quality criteria (Supplementary Table S2). The average quality score was 12.62 (standard deviation = 1.73, range = 10–18). Of these 82 studies, 64 studies were excluded from our meta-analyses due to insufficient data. Data were deemed insufficient if we could not record the number of patients within each outcome of interest according to the three genotype classifications we used (LL, LS and SS). Among the remaining 18 studies, 7 studies were used in the response meta-analysis, and 11 were included in the tolerability meta-analysis.

### 3.1. 5-HTTLPR and Antidepressant Response in Non-MDD Patients

A total of seven studies comprising 535 (range: 39–112) participants investigated the genetic association between the 5-HTTLPR polymorphism and antidepressant response in non-MDD patients (Table 2). Six of the studies primarily included individuals of European ancestry [30,31,32,33,34,35], and one study did not specify the ancestry of the studied population [36]. Clinical diagnoses across studies included OCD (4 studies) [30,31,32,36], GAD (1 study) [33], panic disorder (1 study) [35], and bulimia (1 study) [34]. Most studies used prospective study designs (6 studies) and included at least one SSRI (6 studies). Only one study [33] reported genotyping results for the *SLC6A4* rs25531 polymorphism. Symptom severity scales and thresholds employed varied by study (see Table 2 for details). Three studies [30,32,36] used the Yale–Brown obsessive compulsive scale, while the other studies used the panic disorder severity scale [35], Hamilton anxiety rating scale [33], bulimia investigation test [34], or a single-item three-point severity scale [31].

**Figure 2:**
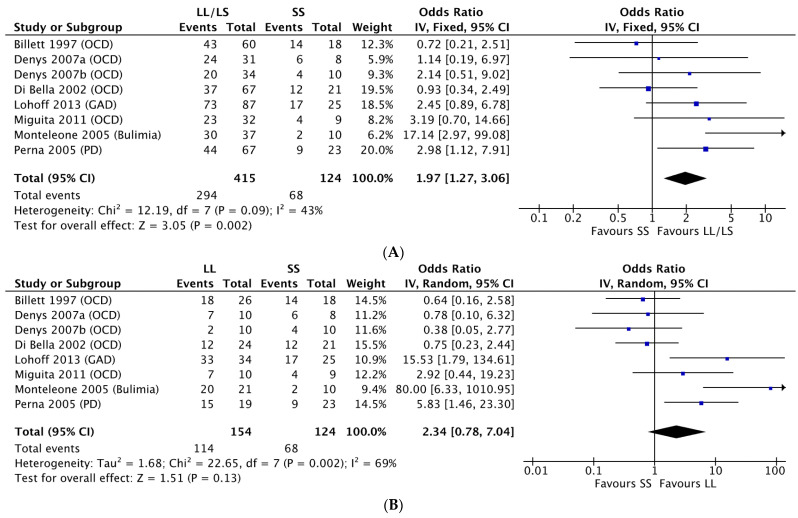

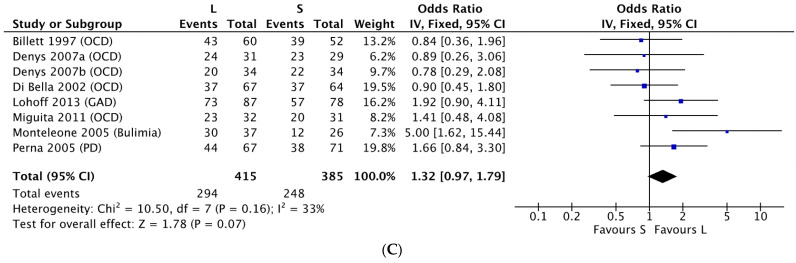
Forest plots of 5-HTTLPR polymorphisms and antidepressant response in all studies by genotype comparisons. (**A**) LL/LS vs. SS; (**B**) LL vs. SS; (**C**) L vs. S. GAD, generalized anxiety disorder; OCD, obsessive compulsive disorder; PD, panic disorder.

A random-effects meta-analysis including all seven studies showed L carriers had greater odds of antidepressant response when compared to carriers of the SS genotype (LL/LS vs. SS: OR = 1.97, 95% CI = 1.27–3.05, *p* = 0.002) (Figure 2). Removal of studies conducted in non-European or unspecified populations revealed similar findings (LL/LS vs. SS: OR = 1.890, 95% CI = 1.19–2.98, *p* = 0.006). Likewise, removal of study data that did not include SSRIs (LL/LS vs. SS: OR = 1.899, 95% CI = 0.721–5.006, *p* = 0.194) or studies that genotyped rs25531 (LL/LS vs. SS: OR 1.879, 95%CI 1.157–3.050, *p* = 0.011) showed similar results found in the full analysis. Only two studies [30,33] reported usable data for non-SSRI, inhibiting stratified analysis. The proportion of females included in a study but not mean age significantly moderated all genotype comparisons (L vs. S: *p* = 0.019; LL vs. SS: *p* = 0.029; LL/LS vs. SS: *p* = 0.016). As the proportion of females included increased, the strength of the association between the 5-HTTLPR polymorphism and antidepressant response increased. Stratified analyses of the four OCD studies did not detect an association, regardless of which genetic model was examined (L vs. S: OR = 0.922, 95% CI = 0.615–1.383, *p* = 0.695; LL vs. SS: OR = 0.803, 95% CI = 0.396–1.629, *p* = 0.544; LL/LS vs. SS: OR = 1.240, 95% CI = 0.688–2.233, *p* = 0.474).

### 3.2. 5-HTTLPR and Antidepressant Tolerability

A total of 2737 (range: 27–1655) antidepressant-treated individuals with a psychiatric disorder were included across the 11 studies (Table 3). Five studies predominantly comprised individuals of European ancestry [8,37,38,39,40], four studies were conducted within the Japanese population [16,41,42,43], and one study was conducted in a North Indian population [17]. The Higuchi et al. [44] study did not specify the ancestry of the studied population. Clinical diagnoses across studies included MDD (nine studies) [8,17,37,39,40,41,42,44], panic disorder (two studies) [16,43], and anxiety disorders (one study) [42]. Most studies used prospective study designs (10 studies) and included at least one SSRI (10 studies). None of the 11 studies reported genotyping results for the *SLC6A4* rs25531 polymorphism, constraining our meta-analyses to bi-allelic 5-HTTLPR associations. Measures of antidepressant tolerability varied by study. The UKU scale was used by three studies [17,41,44] medication discontinuation/drop-out due to an ADR was used by another three studies [16,40,42], while the other studies used the changes in sexual functioning questionnaire [37], global rating of side effect burden [8], or various unspecified self-report measures [38,39,43]. The reported prevalence of ADRs ranged from 5.3–86.1% (Supplementary Table S3).

**Figure 3:**
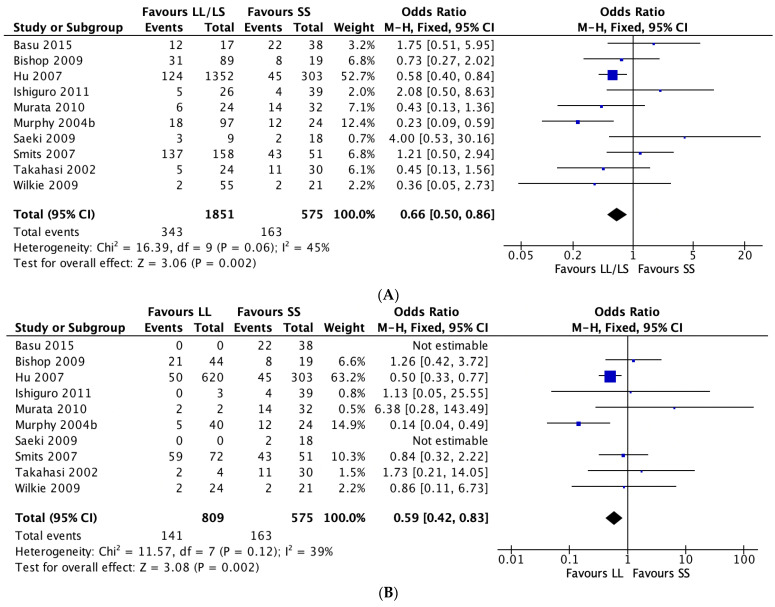

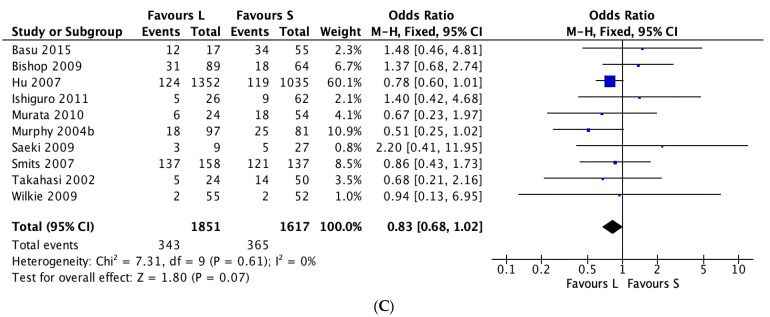
Forest plots of 5-HTTLPR polymorphisms and adverse drug reactions in studies with participants taking SSRIs by genotype comparisons. (**A**) LL/LS vs. SS; (**B**) LL vs. SS; (**C**) L vs. S.

**Figure 4:**
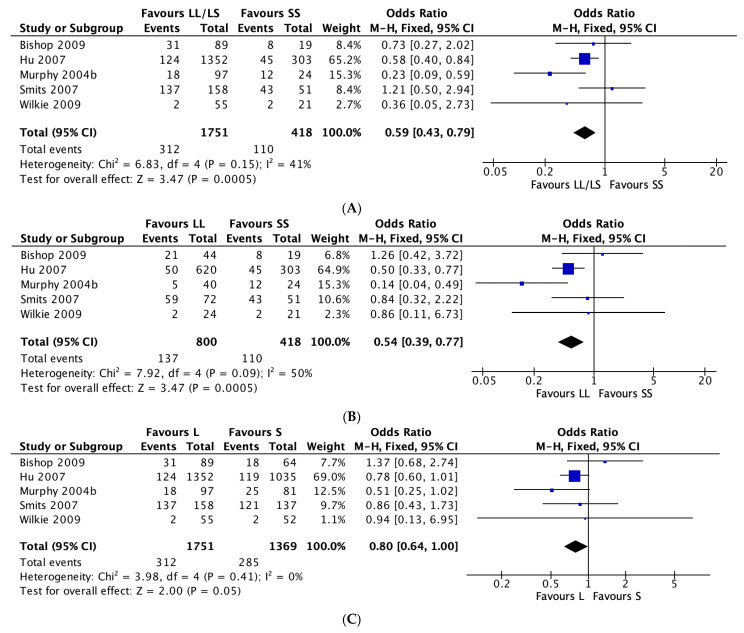
Forest plots of 5-HTTLPR polymorphisms and adverse drug reactions in studies with participants of European background taking SSRIs by genotype comparisons. (**A**) LL/LS vs. SS; (**B**) LL vs. SS; (**C**) L vs. S.

Random-effects pooled ORs showed no significant associations between the three 5-HTTLPR genetic models and antidepressant tolerability (Supplementary Figure S1), and evidence of publication bias was detected when comparing the L vs. S genotypes (p = 0.047) and the LL vs. SS genotypes (p = 0.028) but not the LL/LS vs. SS genotypes (p = 0.061) in the combined study populations (Supplementary Figure S2). Stratified analyses of only SSRI treatment studies, however, showed that L allele carriers reported fewer ADRs relative to SS carriers (LL vs. SS: OR = 0.59, 95% CI = 0.42–0.82, *p* = 0.002; LL/LS vs. SS: OR = 0.64, 95% CI = 0.49–0.84, *p* = 0.001) (Figure 3). Due to the limited number of studies and data, stratification by non-SSRI use was not conducted. Furthermore, when stratified by ancestry, European L carriers taking SSRIs reported fewer ADRs to S carriers (L vs. S: OR = 0.79, 95% CI = 0.64–0.99, *p* = 0.045; LL/LS vs. SS: OR = 0.58, 95% CI = 0.43–0.78, *p* < 0.001) (Figure 4). No associations were detected when the analyses were restricted to studies conducted in the Japanese population (L vs. S: OR = 0.953, 95% CI = 0.51–1.76, *p* = 0.879; LL/LS vs. SS: OR = 0.935, 95% CI = 0.34–2.55, *p* = 0.896; LL vs. SS: OR = 2.43, 95% CI = 0.58–10.14, *p* = 0.221). Likewise, stratified analyses of only MDD studies showed no association (L vs. S: OR = 0.849, 95% CI = 0.691–1.043, *p* = 0.119; LL/LS vs. SS: OR = 0.859, 95% CI = 0.493–1.498, *p* = 0.593; LL vs. SS: OR = 1.045, 95% CI = 0.470–2.320, *p* = 0.915). Sex, age, and ancestry were not significant moderators.

## 4. Discussion

In this systematic review and meta-analysis, the L allele of the 5-HTTLPR polymorphism was shown to be associated with better antidepressant response in patients with non-MDD psychiatric disorders and improved tolerability among individuals with any psychiatric diagnosis. Importantly, these findings were most robust for individuals with European ancestry and those who were treated with SSRIs and may be stronger in females.

### 4.1. 5-HTTLPR and Antidepressant Response

We found that non-MDD L allele carriers had a nearly two-fold greater odds of antidepressant response compared to SS carriers. Our findings concur with the most recent meta-analysis among individuals with MDD that reported 5-HTTLPR L allele carriers of European (OR = 1.36, 95% CI = 1.10–1.68, *p* = 0.005) but not Asian (OR = 0.88, 95% CI = 0.63–1.22, *p* = 0.431) background had greater antidepressant response and remission rates compared to SS carriers, respectively [23]. Collectively, these previous findings and those found in the current study suggest the association between 5-HTTLPR L allele and antidepressant response are unlikely to differ by diagnosis, but the association might be drug class- and ancestry-specific and moderated by sex.

The potential specificity of the association to SSRIs is biologically plausible, given that SSRIs directly bind to the serotonin transporter protein, inhibiting the recycling of serotonin [14]. However, tricyclic antidepressants (e.g., clomipramine) and serotonin-norepinephrine reuptake inhibitors (e.g., venlafaxine, milnacipran) also directly bind to the transporter at equivalent affinities seen for SSRIs [14,45]. As such, the specificity of the association between the 5-HTTLPR polymorphism and SSRI response detected by us and others is more likely an artifact of the small number of studies that have examined this association in the context of non-SSRI treatment. We were unable to derive specific pooled estimates for tricyclic antidepressants or serotonin-norepinephrine reuptake inhibitors due to the small number (less than three) of studies available.

An explanation for the differential ancestry by genotype effect remains unclear. We have previously noted that the frequency of the favorable L allele in people of European ancestry was double that seen in those of Asian ancestry [46], suggesting individuals of Asian ancestry may be at greater genetic risk for SSRI non-response or side effects. However, this explanation is unlikely, given that previous work has shown that SSRI response and tolerability are relatively stable across ethnic groups [47,48]. A more likely explanation is that other variants in population-specific linkage disequilibrium with the 5-HTTLPR polymorphism are the casual variants. Future investigations of the *SLC6A4* and flanking regions are needed to test this hypothesis.

We also detected a moderating effect of sex on the association between the 5-HTTLPR polymorphism and antidepressant response, which suggested that as the proportion of females in a study increased, the association between the L allele and response strengthened. This finding is, in part, supported by the notion that estrogen/estradiol influence serotonin synthesis [49] and increases serotonin transporter expression [50]. This, coupled with established evidence that the L allele is associated with greater serotonin transporter expression relative to the S allele [13], suggests females who carry the L allele may have better antidepressant response rates than males. That said, we were unable to conduct sex-stratified meta-analyses due to the lack of sex-specific data in the included studies, and we cannot rule out other possible explanations (e.g., diagnosis or treatment differences) for this sex effect [51].

### 4.2. 5-HTTLPR and Antidepressant Tolerability

Our pooled findings from 11 studies showed that the 5-HTTLPR polymorphism was also associated with SSRI tolerability, extending and replicating a previous meta-analysis of nine studies that reported a reduced risk of side effects for carriers of the L allele (OR = 0.64, 95% CI = 0.49–0.82, *p* = 0.0005) [47]. Similar to our response results, the association was most robust among individuals of European ancestry and those taking SSRIs, but the limited number of non-European and non-SSRI studies prohibits firm conclusions about the ancestry or drug class specificity of this association.

The mechanism by which the L allele mitigates the increased ADR burden experienced by SS genotype carriers has been hypothesized to be a function of serotonin transporter saturation [52]. Individuals with low expression of the serotonin transporter (i.e., SS genotype carriers) would have greater saturation of the transporter when exposed to an antidepressant, which would elevate central and peripheral levels of serotonin and increase the probability of ADRs [47]. However, this mechanism has not been formally tested to our knowledge.

### 4.3. Limitations

Several caveats of the study should be considered when interpreting the results. First, our pooled estimates were derived from heterogenous studies that included individuals with different psychiatric diagnoses and used varying measures and criteria for determining antidepressant response and presence of an ADR. We statistically mitigated this heterogeneity via the application of a random effects model, but we cannot rule out that this heterogeneity may have resulted in false-negative findings. Second, the clinical phenotype groupings we examined were crude due to the limited number of studies reporting the same specific phenotypes. As the literature expands, meta-analyses for specific antidepressant response phenotypes and ADRs will be possible. Third, dosing information was not routinely or comprehensively reported in the included studies. As a result, we were not able to determine if dose relationships or interactions are present. Fourth, few studies reported associations between 5-HTTLPR and non-SSRIs, inhibiting us from determining the presence or absence of an association. Further work in this area is important, as it has implications for clinical actionability, such as whether switching from an SSRI to a non-SSRI is a reasonable action for individuals with the SS genotype. Likewise, most of the studies included in our meta-analyses did not include the *SLC6A4* rs25531A > G genotype. If we assume the rs25531 genotype can differentially affect the function of the L allele, this would result in misclassification of a portion of L carriers (up to 9% Europeans, 13% East Asians) [53] and may alter the results of our meta-analysis. In fact, no association with antidepressant response was found when Ren and colleagues [23] constrained their meta-analysis to MDD studies that only included the 5-HTTLPR/rs25531 tri-allelic polymorphism, although this may also have been a result of reduced statistical power. Finally, haplotypes in cytochrome P450 genes (*CYP2C19* and *CYP2D6*) associated with the metabolism of most antidepressants [5,6,54] were not accounted for and could explain, in part, the inconsistent findings across studies. Future studies should examine 5-HTTLPR’s association with response and tolerability, while simultaneously accounting for *CYP2D6* and *CYP2C19* genetic variation.

## 5. Conclusions

Given the moderate-to-large pooled ORs detected, our results suggest that the 5-HTTLPR polymorphism might serve as a useful marker for antidepressant response and tolerability in the treatment of psychiatric disorders and may be particularly relevant in clinical care situations where SSRI treatment is being considered for an individual of European ancestry. However, the association between 5-HTTLPR and other alternative treatments (e.g., non-SSRIs, augmentation strategies) remain uncertain due to the paucity of data available. This coupled with methodological and clinical heterogeneity present in the studies conducted to date highlight a need for prospective pragmatic trials of 5-HTTLPR testing to ensure adequate clinical utility and the development of 5-HTTLPR prescribing guidelines to facilitate clinical implementation.

## Figures and Tables

**Table 1:** Summary characteristics of the included studies in the systematic review (N = 82 studies).

Study sample size, Mean (SD)	176 (303.29)
Age, mean (SD)	41.79 (14.45)
Proportion Female, Mean (SD)	51.96 (21.32)
Quality score, Mean (SD)	12.62 (1.73)
Ancestry *, % (N) Studies	
American	1.2 (1)
European	43.9 (36)
East Asian	17.1 (14)
Central/South Asian	1.2 (1)
Near Eastern	4.9 (4)
Not specified	31.7 (26)
**Diagnosis, % (N) Studies**	
Mood disorder ^1^	45.1 (37)
Mood ^1^ and/or anxiety disorder ^2^	2.4 (2)
Mood ^1^, anxiety ^2^ or other ^3^ disorders	1.2 (1)
Anxiety disorder ^2^	19.5 (16)
Chronic tension-type headache	1.2 (1)
Psychotic disorder ^4^	1.2 (1)
Substance-related disorder	6.1 (5)
Eating disorder ^5^	1.2 (1)
Autism spectrum disorder	3.7 (3)
Healthy	1.2 (1)
Other	17.1 (14)
**Antidepressant Used, % (N) Studies**	
Bupropion	2.4 (2)
Bupropion and TCAs	1.2 (1)
Citalopram	11.0 (9)
Desvenlafaxine	1.2 (1)
Escitalopram	9.8 (8)
Fluoxetine	2.4 (2)
Fluvoxamine	6.1 (5)
Milnacipran	1.2 (1)
Paroxetine	11.0 (9)
Sertraline	13.4 (11)
Venlafaxine	2.4 (2)
Mirtazapine and SSRIs	1.2 (1)
Mirtazapine, MAOAs, SNRIs, SSRIs, and TCAs	1.2 (1)
Mirtazapine, Reboxetine, SNRIs, SSRIs, and TCAs	1.2 (1)
SNRIs and SSRIs	3.7 (3)
SNRIs, SSRIs, and TCAs	1.2 (1)
SNRIs, SSRIs, MAOAs, and TCAs	2.4 (2)
SSRIs	15.9 (13)
SSRIs, MAOAs, and TCAs	1.2 (1)
SSRIs and TCAs	8.5 (7)
Various antidepressants (unspecified)	1.2 (1)

* As per PharmGKB biogeographical groups: https://www.pharmgkb.org/page/biogeographicalGroups (accessed on 13 September 2021). ^1^ Mood disorders include: major depressive disorder (MDD), bipolar disorder (BP) type I and II, cyclothymia. Per our inclusion criteria, MDD studies were only included in our tolerability analyses. ^2^ Anxiety disorders include: obsessive compulsive disorder (OCD), generalized anxiety disorder (GAD), panic disorder (PD), social anxiety disorder (SAD), post-traumatic stress disorder (PTSD); ^3^ Other was not specified. ^4^ Psychotic disorders include: schizophrenia, schizoaffective disorder. ^5^ Eating disorders include: bulimia. Abbreviations used: MAOAs, monoamine oxidase inhibitors; SD, standard deviation; SNRIs, selective norepinephrine reuptake inhibitors; SSRIs, selective serotonin reuptake inhibitors; TCAs, tricyclic antidepressants.

**Table 2:** Characteristics of studies (*n* = 7) included in the meta-analysis of antidepressant treatment response in patients with psychiatric disorders other than MDD.

STUDY (Author et al.)	STUDY Design	N	Age [Mean, Years]	Sex [Female (%)]	Ancestry	Diagnosis	Antidepressant (s) Used	Other Drug (s) Used	*SLC6A4* rs25531 Tested?	5-HTTLPR Genotype Frequencies	Phenotype (s) Measurement	Quality Score *
Billett et al. (1997) [31]	Retrospective Case-Control Study	72	36.3	53	European	OCD	SSRIs (Fluoxetine, Clomipramine, Fluvoxamine, Paroxetine, Sertraline)	Not Available	No	SS = 23% SL = 44% LL = 33%	Symptom severity had decreased by at least 25% (Measured with a 3-point scale)	11
Denys et al. (2007) [30]	Prospective Parallel-group Study	39	33.2	61	European	OCD	Paroxetine	Not Available	No	SS = 20% SL = 54% LL = 26%	YBOCS (≥25% reduction from baseline)	13
		44	33.2	61	European	OCD	Venlafaxine	Not Available	No	SS = 23% SL = 54% LL = 23%	YBOCS (≥25% reduction from baseline)	
Di Bella et al. (2002) [32]	Prospective Case-Control Study	88	33.37	50	European	OCD	Fluvoxamine	Not Available	No	SS = 24% SL = 49% LL = 27%	YBOCS (>35% reduction from baseline)	16
Lohoff et al. (2013) [33]	Prospective Cohort Study	112	>18 years	Not Available	European (72%)	GAD	Venlafaxine	Benzodiazepine Anxiolytics, Hypnotics	Yes	SS = 22% SL = 47% LL = 31%	HAM-A (50% reduction)	12
Miguita et al. (2011) [36]	Prospective Cohort Study	41	35	44	Not Available	OCD	Clomipramine, Tricyclics, SSRIs	Not Available	No	SS = 22% SL = 54% LL = 24%	Y-BOCS Score (>40% reduction from baseline)	12
Monteleone et al. (2005) [34]	Prospective Naturalistic Study	47	>18 years	100	European	Bulimia	SSRIs	Not Available	No	SS = 21% SL = 34% LL = 45%	Bulimia Investigation Test (>50% reduction in binge purging)	11
Perna et al. (2005) [35]	Prospective Cohort Study	92	34	55	European	PD	Paroxetine	Not Available	No	SS = 26% SL = 53% LL = 21%	PDSS-total scores (50% reduction from baseline)	13

* The quality score ranges from 0–26 with higher scores representing higher quality. See Table S2 for detailed information on the specific quality metrics for each study. Abbreviations used: SS, two copies of the short allele; SL, short and long allele; LL, two copies of the long allele; GAD, generalized anxiety disorder; HAM-A, Hamilton rating scale for anxiety; MDD, major depressive disorder; OCD, obsessive compulsive disorder; PDSS, panic disorder severity scale; PD, panic disorder; YBOCS, yale–brown obsessive compulsive scale.

**Table 3:** Characteristics of studies (*n* = 11) included in the meta-analysis on antidepressant tolerability.

Study (Author et al.)	Study Design	N	Age [Mean, Years]	Sex [Female (%)]	Ancestry	Diagnosis	Antidepressant (s) Used	Other Drug (s) Used	*SLC6A4* rs25531 Tested?	5-HTTLPR Genotype Frequencies	Phenotype (s) Measurement	Quality Score *
Basu et al. (2015) [17]	Prospective Cohort Study	55	35	42	North Indian	MDD	Escitalopram	Anxiolytics, Sedatives, Hypnotics	No	SS = 69% SL = 31% LL = 0%	UKU scores (all side effects recorded irrespective of severity and degree of association)	14
Bishop et al. (2009) [37]	Prospective Cohort Study	115	29.2	76	European (92%)	MDD	SSRIs (Citalopram, Escitalopram, Fluoxetine, Paroxetine, Sertraline)	Not Available	No	SS = 18% SL = 42% LL = 40%	Changes in sexual functioning questionnaire (CSFQ) (scores lower than 47 for males and 42 for females indicate decreased sexual desire or function)	13
Higuchi et al. (2009) [44]	Prospective Cohort Study	80	52.4	65	Not Available	MDD	Milnacipran	Brotizolam	No	SS = 65% SL = 34% LL = 1%	UKU scores (nausea) (adverse events were recorded if the score was greater than 1 and were not present before treatment)	15
										SS = 64% SL = 35% LL = 1%	UKU scores (sweating) (adverse events were recorded if the score was greater than 1 and were not present before treatment)	15
Hu et al. (2007) [8]	Prospective Case-Control Study	1655	42	62	European (79.9%)	MDD	Citalopram	Not Available	No	SS = 18% SL = 44% LL = 38%	Global rating of side effect burden (GRSEB) (score of 4 or greater indicated increased adverse effects)	11
Ishiguro et al. (2011) [16]	Prospective Cohort Study	65	36	65	Japanese	PD	Paroxetine	Brotizolam, Lorazepam	No	SS = 60% SL = 35% LL = 5%	No. of dropouts due to ADRs	12
Murata et al. (2010) [42]	Prospective Cohort Study	56	45.9	57	Japanese	MDD, Anxiety Disorder, or others (e.g., pain disorder)	Paroxetine	Tandospirone, Benzodiazepines, Zolpidem, Zopiclone	No	SS = 57% SL = 39% LL = 4%	Paroxetine discontinuation-emergent events (at least 1 qualitatively new symptom within 7 days after stopping medication)	14
Murphy et al. (2004) [40]	Prospective Cohort Study	124	72	50	European (94%)	MDD	Mirtazapine	Not Available	No	SS = 25% SL = 44% LL = 31%	No. of discontinuations as a result of at least 1 adverse events	12
		122	72	52	European (89%)	MDD	Paroxetine	Not Available	No	SS = 20% SL = 47% LL = 33%	No. of discontinuation as a result of at least 1 adverse events	
Saeki et al. (2009) [43]	Prospective Cohort Study	27	34.3	78	Japanese	PD	Paroxetine	Brotizolam, Lorazepam	No	SS = 67% SL = 33% LL = 0%	Self-report (experienced at least 1 symptom including drowsiness or abnormal sensation)	12
Smits et al. (2007) [39]	Retrospective Cohort Study	214	48.48	70	European	MDD	SSRIs (Paroxetine, Fluoxetine, Fluvoxamine, Sertraline, Citalopram)	Not Available	No	SS = 24% SL = 41% LL = 33%	Complaints made in face-to-face interview (at least 1 adverse event that began after medication use)	15
Takahasi et al. (2002) [41]	Prospective Cohort Study	54	51.52	59	Japanese	MDD	Fluvoxamine	Brotizolam	No	SS = 55% SL = 36% LL = 7%	UKU score (recorded patients with nausea according to scale criteria)	12
Wilkie et al. (2009) [38]	Prospective Cohort Study	166	43.42	69	European	MDD	Paroxetine, Imipramine, Lofepramine, Phenelzine	Not Available	No	SS = 28% SL = 41% LL = 32%	Adverse events (not specifically defined)	13

* The quality score ranges from 0–26, with higher scores representing higher quality. See Table S2 for detailed information on the specific quality metrics for each study. Abbreviations used: SS, two copies of the short allele; SL, short and long allele; LL, two copies of the long allele; MDD, major depression disorder; PD, panic disorder; QIDS-C score, quick inventory of depressive symptomatology; UKU, udvalg for kliniske undersogelser Score.

## Data Availability

Data are available from the cited studies included in this review.

## Supplementary Materials

The following are available online at https://www.mdpi.com/article/10.3390/jpm11121334/s1, Table S1: Characteristics of studies (*n* = 82) included in the systematic review and meta-analysis of *5-HTLLPR* and antidepressant response and tolerability in patients with psychiatric disorders; Table S2: Summary of quality score of included studies. Table S3: Reported prevalence of ADRs among studies in the tolerability meta-analysis. Summary of quality score of included studies. Figure S1: Forest plots of 5-HTTLPR polymorphisms and adverse drug reactions in all studies by genotype comparisons (LL/LS vs. SS, LL vs. SS, L vs. S). Figure S2: Funnel plots of 5-HTTLPR polymorphisms and adverse drug reactions in all studies by genotype comparisons (LL/LS vs. SS, LL vs. SS, L vs. S).
