# Supplementary material for: Serotonin Transporter Genetic Variation and Antidepressant Response and Tolerability: A Systematic Review and Meta-Analysis
Source: J Pers Med. 2021 Dec 9;11(12):1334. doi: 10.3390/jpm11121334 (PMC8707702; doi:10.3390/jpm11121334)
Supplement: Supplementary file 1 [file jpm-11-01334-s001.zip › jpm-1424887-SI.pdf]

Table S1. Characteristics of studies (n = 82) included in the systematic review and meta-analysis of *5-HTLLPR* and antidepressant response and tolerability in patients with psychiatric disorders.

Study (Author *et al.*)	Pubmed ID	Study Design	N	Age [Mean Years]	Sex [Female (%)]	Ancestry/ Race	Diagnosis	Antidepressant(s) Used	Other Drug(s) Used	*SLC6A4* Variants Tested	Phenotype(s)	Phenotype(s) Measurement	Main Findings	Quality Score
Abdelmalik *et al.* (2009) [1]	18983505	Prospective Cohort Study	19	44.5	58	Not Available	Mood Disorder and/or Anxiety	Paroxetine	Depakine, Levothyroxin, Omeprazole	5-HTTLPR/ rs25531	Platelet Function	Bleeding Severity Scores	Paroxetine impairs platelet function by decreasing the levels of platelet serotonin and platelet β-TG. These effects are mediated by 5HTTLPR, with pronounced effects in patients without La alleles	11
Akcali *et al.* (2008) [2]	18688140	Prospective CaseControl Study	264 CTTH=126 Healthy Controls=138	CTTH=38 Healthy Controls=23	CTTH=87 Healthy Controls=85	Not Available	CTTH	Amitriptyline, Citalopram, Sertraline	Not Available	5-HTTLPR, VNTR	Improvement in headache symptoms	VAS Scores	No statistically significant association between genotype and study outcome	12
AlOlaby *et al.* (2017) [3]	28242040	Prospective PlaceboControlled Study	51	46.1	11.8	Not Available	Children with Fragile X syndrome	Sertraline	Not Available	5-HTTLPR	Cognitive Improvement	MSEL Scores, CGI-I, VAS Scores, SPM Scores	Patients with the LL genotype showed a significant improvement in social participation scores relative to the placebo group	12
Basu *et al.* (2015) [4]	26261165	Prospective Cohort Study	55	35	42	North Indian	MDD	Escitalopram	Anxiolytics, sedatives and hypnotics allowed	5-HTTLPR	Depression Severity and side effects	MADRS, CGI, UKU Scale	No statistically significant association between genotype and study outcome	14
Baumer *et al.* (2006) [5]	16945343	Retrospecti ve Case Control Study	52	15.1	31	Caucasian (88.2%), Hispanic (5.9%), Multiracial (5.9%)	Bipolar and Subsyndromal Participants	Fluoxetine, Paroxetine, Sertraline, Citalopram, Fluvoxamine, Escitalopram,	Atypical antidepressants: Bupropion, Venlafaxine, Trazadone, Serzone	5-HTTLPR	AIM Status	YMRS	All 3 participants with the SS genotype responded negatively to antidepressants	17
Billett *et al.* (1997) [6]	9322235	Retrospecti ve CaseControl Study	144	36.3	53	Not Available	OCD	Fluoxetine, Clomipramine, Fluvoxamine, Paroxetine, Sertraline	Not Available	5-HTTLPR	Severity of OCD Symptoms	3 Point Rating Scale	No statistically significant association between genotype and study outcome	11

Bishop *et al.* (2009) [7]	19204908	Naturalistic Prospective Study	115	29	76	Caucasian (92%), Hispanic (3%), Asian (2%), African American/Other (2%)	MDD	Citalopram, Escitalopram, Fluoxetine, Paroxetine, Sertraline, Fluvoxamine	Not Available	5-HTTLPR	Sexual WellBeing	CSFQ	Females with the LL genotype who were taking oral contraceptive medication had statistically significant lower CSFQ scores than makes with the LL genotype. No relationship was observed in females who were not taking oral contraceptives	13
Bloch *et al.* 2010 [8]	19995670	Prospective Placebo Controlled Study	32	41	25	Northern Israel Population	Schizophrenia or Schizoaffective Disorder	Bupropion	Placebo	5-HTTLPR	Smoking Behavior and Schizophrenia Severity	FTND, BPRs, PANSS	Male subjects with SS or SL genotype showed a reduction in cigarette consumption as a result of treatment	12
de Aguiar Ferreira *et al.* (2009) [9]	18534687	Retrospecti ve Case Study	112	39	71	Caucasian (Brazilian)	Bipolar Disorder	Venlafaxine, SSRIs, Tricyclics	Mood stabilizers: Lithium, Sodium Divalproate, Carbamazepin e, Oxcarbazepin e, Lamotrigine	5-HTTLPR	AAM Status	Interviewed by psychiatrists and followed the DSM-IV criteria	S carriers (LS/SS genotypes) are more prone to have a manic/hypomanic episode associated with antidepressants	11
Denys *et al.* (2007) [10]	17503984	Prospective Parallelgroup Study	91	33.2	61	European Descent	OCD	Venlafaxine, Paroxetine	Not Available	5-HTTLPR	Antidepressan t side effects	UKU Scale	Response in venlafaxine treated OCD patients is associated with the SL genotype	13
Di Bella *et al.* (2002) [11]	12082589	Prospective CaseControl Study	372	33.37	52	Caucasian (Italian Descent)	OCD	Fluvoxamine	Not Available	5-HTTLPR	Clinical Outcomes	Treatmentemergent adverse effects were assessed in each person visit	Participants with the SS genotype developed new or worsening insomnia and agitation	16

Dombrovski *et al.* (2010) [12]	19996755	Prospective Cohort Study	92	81 (Estimate)	62	75% Caucasian	Dementia	Citalopram	Risperidone	5-HTTLPR	Clinical Outcomes	NBRS, BPRS, UKU, MMSE, CIRS-G	Participants with the SS genotype had the shortest time-totreatment discontinuation. Excluding African American participants, participants with the SS or SL genotypes predicted greater adverse effects of citalopram. Participants with the SS or SL genotype taking risperidone predicted poorer early response of psychosis symptoms	13
Durham *et al.* (2004) [13]	12955294	Prospective Placebo-Controlled Study	217	69	53	Caucasian (95.4%), Black (2.8%), Other (1.38%), Asian (0.46%)	MDD	Sertraline	Not Available	5-HTTLPR	Clinical Outcomes and Response Time	HAM-D, CGI-I	There was a significant increase in number of CGI-I responders in the LL genotype group at weeks 1 and 2	11
Frye *et al.* (2015) [14]	25611077	Retrospecti ve CaseControl Study	295	Not Availab le	59	Caucasian	Bipolar	SSRIs	Not Available	5-HTTLPR, 5-HTTLPR/rs 25531, VNTR	AIM status	DSM-IV structured interviews	Bipolar I subtype was the only clinical risk factor associated with AIM	9
Garfield *et al.* (2014) [15]	24021217	Prospective PlaceboControlled Study	177	71	67	Not Available	GAD	Escitalopram	Low-dose benzodiazepines if already using for at least 2 months	HTTLPR/rs 25531	Clinical Outcomes	UKU	Greater dry mouth was observed in participants with the low expressing genotype. Greater sexual desire was observed in patients with the high expressing genotype	13
Gressier *et al.* (2014) [16]	25257397	Naturalistic Prospective Study	103	Nonmenopausal: 36.6 Menopausal: 60.8	72	Caucasian	Unipolar or Bipolar	Paroxetine, Fluoxetine, Escitalopram, Citalopram, Sertraline	Anxiolytics and hypnotics at minimal doses	5-HTTLPR	Clinical Outcomes	HAMD-17, CGI-I	In non-menopausal women, the L allele was associated with high antidepressant efficacy.	10

Han *et al.* (2008) [17]	18197080	Prospective Cohort Study	225	Not Availab le	0	Korean	Smokers	Bupropion	Not Available	5-HTTLPR, VNTR	Abstinence of Smoking	Assessed during visits	Participants with the LL genotype showed a higher abstinence rate than other participants	11
Higuchi *et al.* (2009) [18]	19649213	Prospective Cohort Study	80	52.4	65	Not Available	MDD	Milnacipran	Brotizolam	5-HTTLPR, VNTR	Antidepressan t side effects	UKU	No statistically significant between genotype and study outcome	15
Hougardy *et al.* (2008) [19]	18279474	Naturalistic Prospective Study	43	48.4	74	Not Available	Patients taking Paroxetine for >4 weeks	Paroxetine	Not Available	5-HTTLPR	Bleeding time	PFA, Trough paroxetine levels, complete blood count	No statistically significant association between genotype and study outcome	11
Hu *et al.* (2007)[20]	17606812	Prospective CaseControl Study	2626	Depress ed: 42.4 Control: 51.9	58	Caucasian (81.6%), Black (14.2%), Other (6%)	MDD	Citalopram	Not Available	5-HTTLPR	Clinical Outcomes	QIDS-C Score	The LA allele was associated with adverse effect burden in the entire sample	11
Huezo-Diaz *et al.* (2009) [21]	19567893	Prospective Cohort Study	810	Range:1 9-72	63.5	Caucasian (European Descent)	MDD	Nortriptyline, Escitalopram	Hypnotics	5-HTTLPR, 5-HTTLPR/rs 25531, STin2	Clinical Outcomes	MADRS, HRSD-17, selfreport 21 item BDI	Among participants taking Escitalopram, those carrying the long-allele improved more than the shortallele carriers	14
Ishiguro *et al.* (2011) [22]	21688171	Prospective Cohort Study	65	36	65	Japanese	PD	Paroxetine	Brotizolam, and Lorazepam	5-HTTLPR	Clinical Outcomes	PAS	Participants with the SS genotype showed improved clinical response after 2 weeks. No significant difference in clinical response was observed at 4 weeks	12
Joyce at al. (2005) [23]	15856721	Prospective Cohort Study	195	31	57	Not Available	MDD with Bipolar II, Melancholia, Atypical, Recurrent	Fluoxetine, Nortriptyline	Oral contraceptive, hypnotics	5-HTTLPR	Diurnal Variation	Amino acid ratio and revised spectrophotofl uorimetric technique	Participants with reversed diurnal variation have significantly higher Trp:LNAA ratios, and are more likely to have the s allele of the 5-HTTLPR	10

Kato *et al.* (2006) [24]	16874005	Prospective Cohort Study	100	43.7	44	Japanese	MDD	Paroxetine, Fluvoxamine	Not Available	5-HTTLPR	Clinical Outcomes	HAM-D	There were statistically significant greater number of responders among the L allele carriers at weeks 4 and 6	13
Kenna *et al.* (2009) [25]	19032576	Prospective Cohort Study	15	44.1	20	Not Available	Alcoholdependent	Sertraline	Ondansetron	5-HTTLPR	Alcohol Dependency Severity	Alcohol Consumption using TLFB	Participants taking Ondansetron with the LL genotype showed a significant improvement in drinking outcomes	12
Kenna *et al.* (2014) [26]	25212749	Prospective PlaceboControlled Study	49	43	39	Caucasian (53.1%), AfricanAmerican (28.6%), Hawaiian or Alaskan Multiethnic (10.2%), Hispanic (6.1%), American Indian (2%)	Non-treatment seeking alcohol dependent individuals	Sertraline	Ondansetron	5-HTTLPR, VNTR	Alcohol Dependency Severity	Alcohol Consumption using TLFB	No statistically significant association between genotype and study outcome.	15
Kranzler *et al.* (2011) [27]	21192139	Prospective PlaceboControlled Cohort Study	134	Range:18-65	19.5	Not Available	Alcohol Dependency	Sertraline	Not Available	5-HTTLPR	Alcohol Dependency Severity	Alcohol Consumption using TLFB, SIP, GGTP	In the LL genotype group, the effects of medication group varied by age of onset.	12
Kranzler *et al.* (2012) [28]	21981418	Prospective Case-Controlled Study	134	47.5	19	Caucasian (92%)	Alcohol Dependency	Sertraline	Not Available	5-HTTLPR	Alcohol Dependency Severity	Alcohol Consumption using TLFB	Late-onset alcoholic participants taking Sertraline with the LL genotype had fewer drinking days than the placebo group at the 3-month follow-up	10
Kranzler *et al.* (2013) [29]	23145795	Prospective Cohort Study	134	47.5	19	Caucasian (European Descent) (92%)	Alcohol Dependency	Sertraline	Not Available	5-HTTLPR	Alcohol Dependency Severity and Daily Mood	Daily surveys through IVR technology	Anxiety was a key moderator of the pharmacogenetic effects on drinking severity outcomes	12

Kronenberg *et al.* (2007) [30]	18315446	Prospective Cohort Study	74	7-18	45	Ashkenazi Background	MDD	Citalopram	Not Available	5-HTTLPR	Clinical Outcomes	K-SADS-PL, CGI-S, CGI-I, CDRS-R, SCARED	Participants with the SS genotype showed a poorer improvement in depressive symptoms, exhibited lower rates of agitation and had consistently higher scores of suicidality compared to the participants with SL or LL genotype	12
Lanctôt *et al.* (2010) [31]	20515362	Prospective Cohort Study	90	39.9	44	Caucasian (52.2%), Other (34.4%), Asian (13.3%)	Traumatic Brain Injury with Major Depressive Episode	Citalopram	Not Available	5-HTTLPR, 5-HTTLPR/rs 25531	Clinical Outcomes	HAMD	The SS genotype was significantly predictive of an adverse drug event index, accounting for 6.9% of the variance. The SL genotype had a higher adverse event index value compared to the SS group	12
Lee *et al.* (2010) [32]	20664233	Prospective Cohort Study	84	44.6	60	Korean	MDD	Venlafaxine	No other psychotropic drugs permitted	5-HTTLPR, 5-HTTVNTR	Clinical Outcomes	HAM-A, BAI, HAM-D, MADRS, BDI, Toronto side effect scale	Participants with the LL and LS genotype taking venlafaxine was associated with treatment response at week 4	13
Lee *et al.* (2018) [33]	29030421	Prospective PlaceboControlled Study	301	62.7	38	South Korean	Patients with acute stroke (within 21 days of onset). Patients with history of depression were excluded.	Escitalopram	Not Available	5-HTTLPR, 5-HTTVNTR	Depression Symptoms and Cognitive Function	mRS, NIHSS, MADRS, MoCA	No statistically significant association between genotype and study outcome	12
Lenze *et al.* 2010 [34]	21105279	Prospective PlaceboControlled Study	125	71.5	62	Caucasian	GAD	Escitalopram	Benzodiazepines	5-HTTLPR/rs 25531	Clinical Outcomes	CGI-I, PSWQ, HAM-A	Escitalopram had no efficacy in the La- group but had moderate efficacy in the La+ group	13
Liu *et al.* (2018) [35]	29310115	Prospective CaseControl Study	244	45	60	Chinese	Patients with Globulus Pharyngeas with Anxiety and Depression	Amitriptyline, Paroxetine	Not Available	5-HTTLPR	Clinical Improvements and upper esophageal sphincter pressure	HAM-A, HAM-D	There was a significant association between the SS genotype and response to antidepressant treatment	14

Lohoff *et al.* (2013) [36]	22907732	Prospective Cohort Study	112	>18	Not Available	Caucasian (72%)	GAD	Venlafaxine	Benzodiazepine Anxiolytics, and Hypnotics	5-HTTLPR/rs25531	Clinical Outcomes	HAM-A, CGI	Caucasian subjects who had the La/La genotype showed a significant reduction in HAM-A scores and better treatment response compared with S carriers at 6 months	12
Mandelli *et al.* (2009) [37]	19332357	Prospective Cohort Study	86	45.7	51	Caucasian (Italian population)	Bipolar I, Bipolar II, Cyclothymic Disorder	Conventional antidepressants	Mood Stabilizers, antipsychotics, sedative/anxiolytic drugs	5-HTTLPR, 5-HTTVNTR, 5-HTTLPR/rs25531	Clinical Outcomes	HAMD	A high harm avoidance impaired outcome was associated with carriers of the S allele	13
Maron *et al.* (2009) [38]	19272758	Prospective PlaceboControlled Study	135	31.1	68	Not Available	MDD	Escitalopram	Hormonal contraceptives, Zolpidem, Zopiclone	5-HTTLPR, 5-HTTLPR/rs25531	Clinical Outcomes	MADRS, HAM-D, CGI, BDI	Patients carrying the S allele may have increased risk for headaches, induced by escitalopram.	14
Masoliver *et al.* (2006) [39]	16395126	Prospective CaseControl Study	288	Not Available	Not Available	Not Available	Bipolar or Unipolar Disorder	Tricyclics, Monoamine oxidase inhibitors, SSRIs, Venlafaxine	Lithium	5-HTTLPR, 5-HTTVNTR	History of AIM	DSM-IV Criteria	Association analysis showed a higher rate of the SS genotype among patients with a history of AIM	11
Miguita *et al.* (2011) [40]	21625751	Prospective Cohort Study	41	20+	44	Not Available	OCD	Tricyclics, SRIs	Clomipramine	5-HTTLPR, 5-HTTVNTR	Clinical Outcomes	Y-BOCS	No statistically significant between genotype and study outcome	12
Monteleone *et al.* (2005) [41]	15940301	Prospective Naturalistic Study	47	>18 years	100	Caucasian	Bulimia	Fluoxetine, Paroxetine, Sertraline, Fluvoxamine, Citalopram, Escitalopram	Not Available	5-HTTLPR	Bulimia Severity	The eating disorder inventory-2, the bulimia investigation test Edinburgh, MADRS	Participants with the SS genotype were significantly more likely to be in the non-responder group than the participants with an SL or LL genotype	11
Murata *et al.* (2010) [42]	20075642	Prospective Cohort Study	56	45.9	57	Japanese	MDD, Anxiety Disorder, or pain disorder	Paroxetine	Tandospirone, Benzodiazepines, NonBenzodiazepines	5-HTTLPR, 5-HTTVNTR	Paroxetine Discontinuation Emergent Events	Qualitative Assessment of Symptoms	No statistically significant between genotype and study outcome	14

Murphy *et al.* (2004) [43]	15520364	Prospective Cohort Study	246	>65	52	Caucasian (92%)	MDD	Mirtazapine, Paroxetine	Not Available	5-HTTLPR	Clinical Outcomes	Adverse Event rating using a 3-point scale	Carriers of the S allele taking Paroxetine experienced more severe adverse events and had more discontinuations. Carriers of the S allele taking Mirtazepine had less severe adverse events and fewer discontinuations	12
Mushtaq *et al.* (2012) [44]	21962566	Prospective Cohort Study	226	41.4	55	Not Available	PTSD	Sertraline	Not Available	5-HTTLPR	Clinical Outcomes, Adverse Events	CAPS, IES[R], CGI-S	The improvement slope at 12 weeks was significantly steeper in the group with the LL genotype compared to the LS and SS	15
Najjar *et al.* (2015) [45]	26262902	Prospective Cohort Study	44	13.4	33	Caucasian (60%), African American (18%), Hispanic (16%), Asian (4%), African American/Hispanic (2%)	ASD	Escitalopram	Not Available	5-HTTLPR, 5-HTTLPR/rs25531	Sameness and Irritability Symptoms	RBS-R, ABCCV	No statistically significant association between genotype and study outcome	12
Ng *et al.* (2006) [46]	16580768	Prospective Cohort Study	45	41	38	Chinese (66.7%), Caucasian (33.3%)	MDD	Sertraline	Hypnotics (Zopiclone), Anxiolytics (Lorazepam)	5-HTTLPR	Clinical Outcomes	HDRS, CGI, LUNSERS	No statistically significant association between genotype and study outcome	11

Ng *et al.* (2013) [47]	24014145	Prospective Cohort Study	106	44	60	Caucasian (76%), Han Chinese (24%)	MDD	Escitalopram, Venlafaxine	Not Available	5-HTTLPR, 5-HTTVNTR	Clinical Outcomes	HDRS, UKU	Participants with the LL genotype was associated with significantly greater HDRS score reduction compared to the SS genotype among Caucasian subjects taking Escitalopram. Response rates were significantly higher for participants with the LL genotype than LS or SS.	11
Ng *et al.* (2016) [48]	27023264	Prospective Cohort Study	35	>18	Not Available	Not Available	MDD	Desvenlafaxine	Not Available	5-HTTLPR	Clinical Outcomes	HDRS, CGI, UKU	No statistically significant between genotype and study outcome	13
Owley *et al.* (2010) [49]	20020537	Prospective Cohort Study	58	9.75	17	Caucasian (83%), African American (10%), Asian (3%), Hispanic (3%)	ASD	Escitalopram	Not Available	5-HTTLPR	Clinical Outcomes	ABC-CV	No statistically significant between genotype and study outcome	13
Ozbek *et al.* (2014) [50]	25518026	Prospective Cohort Study	69	Range:21-59	0	Caucasian (Turkish)	Premature Ejaculation	Paroxetine	Not Available	5-HTTLPR	Clinical Outcomes	Intravaginal Ejaculatory Latency Time, International Index of Erectile Function Scores	Participants with the S allele were significantly more likely to belong to the responder group	12
Park *et al.* (2016) [51]	26508020	Prospective Cohort Study	24	39.5	58	Korean	PD	Escitalopram, Paroxetine, Sertraline, Venlafaxine	Not Available	5-HTTLPR	PD Severity	PDSS	Unable to analyze the relationship of genotype and outcome because of low statistical power	10
Perlis *et al.* (2003) [52]	14573314	Prospective Cohort Study	36	36	53	Caucasian (nonLatino)	MDD	Fluoxetine	Not Available	5-HTTLPR	Clinical Outcomes	HAMD-17	Patients with the S allele are at higher risk for developing insomnia or agitation with fluoxetine treatment	12

Perna *et al.* 2005 [53]	16034444	Prospective Cohort Study	92	34	55	Italian Descent	PD (with or without agoraphobia)	Paroxetine	Not Available	5-HTTLPR	PD Severity	PASS, FQ	Good responders were significantly more frequent among female LL and LS carriers	13
Peters *et al.* (2016) [54]	26303700	Retrospecti ve PlaceboControlled Cohort Study	175	80	46	Caucasian (70%), African American (16.6%), Other (6.9%), Asian (3.4%), Pacific Islander/Hawaiian (1.1%)	Alzheimer's Dementia	Citalopram	Placebo	5-HTTLPR	Clinical Outcomes	CGI-I, Hamilton Depression Scale	No statistically significant between genotype and study outcome	12
Popp *et al.* (2006) [55]	16515395	Retrospecti ve Cohort Study	109	49	61	Caucasian (97%), Turkish (1%), Libyan (1%)	MDD	Mirtazapine, Citalopram, Escitalopram, Sertraline, Venlafaxine, Paroxetine, Amitriptyline, Reboxetine, Trimipramine, Fluoxetine, Clomipramine, Opipramol, Tranylcypromine	Doxepin,	5-HTTLPR, 5-HTTVNTR	Clinical Outcomes	CGI	Patients predominantly on HTT-blocking antidepressants with the SS genotype suffered more frequently from side effects	13
Putzhammer *et al.* (2005) [56]	15322730	Prospective Cohort Study	62	Mean between 40-45	69	Not Available	MDD	SSRIs, Tricyclics	Not Available	5-HTTLPR	Motor Activity	Mini-Motionlogger Actograph Basic	Nighttime motor activity was significantly higher in participants taking SSRIs with the LL genotype in comparison to the SL and SS genotype	13
Quaak *et al.* (2012) [57]	21658141	Prospective PlaceboControlled Study	214	51.5	51	Not Available	Current Daily Smokers	Bupropion, Nortriptyline	Not Available	5-HTTLPR, 5-HTTVNTR, 5-HTTLPR/rs 25531	Cessation of Smoking	Urinary Nicotine Values, Abstinence Rates	Participants with the LS or LL genotype who were treated with Bupropion showed increased levels of abstinence. Effects were not significant for those with the SS genotype	11

Rahikainen *et al.* (2017) [58]	28608626	Retrospective Matched Case Study	633 (Deceased Patients)	47	55.4	Not Available	Autopsies positive for Citalopram	Citalopram	Not Available	5-HTTLPR/rs25531	Type of Death (Suicide)	Death Classification (violent of non-violent suicide)	There was a significant association between males with the SS genotype and violent suicide. The SS genotype was associated with repeated suicide attempts and violent suicide attempts	12
Rotberg *et al.* (2013) [59]	23510446	Prospective/ Retrospective Cohort Study	83	13.9	55	Not Available	Depression or Anxiety Disorder of Moderate Severity	Citalopram	Not Available	5-HTTLPR	Clinical Outcomes	CGI	Participants with the S allele had lower response rates	16
Rousseva *et al.* (2003) [60]	12746735	CaseControl Study	305	26	44	Caucasian	Bipolar Affective Disorder	Tricyclics, SSRIs, Venlafaxine, Mirtazapine, Moclobemide, Nomifensine, Trazadone, Amineptine	Not Available	5-HTTLPR	History of AIM	Evaluated according to DSM-IV criteria	No statistically significant assocation between genotype and study outcome	13
Saeki *et al.* (2009) [61]	19259652	Prospective Cohort Study	27	34.3	78	Japanese	PD	Paroxetine	Brotizolam, and Lorazepam	5-HTTLPR	PD Severity, Plasma concentration of Paroxetine	PAS	There was a significant negative correlation between the percent reduction in PAS score and the plasma concentration of PAX in participants with the SS genotype	12
Sahraian *et al.* (2013) [62]	24130607	Prospective Cohort Study	104	Range: 18-65	75	Iranian (Fars background)	MDD	Citalopram	Alprazolam	5-HTTLPR	Clinical Outcomes	UKU, Selfreport Antidepressant Side Effect Checklist	L carriers were associated with an improved response to citalopram when compared to carriers of the SS genotype	14
Saiz-Rodríguez *et al.* (2018) [63]	29136336	Prospective Cohort Study	46	23	48	Caucasian	Healthy Volunteers	Sertraline	No other drugs allowed	5-HTTLPR	Plasma Concentration of Sertraline and ADRs	Liquid chromatographic/tandem mass spectrometric method	No statistically significant association between genotype and study outcome	11
Salem *et al.* (2017) [64]	27679962	Prospective CaseControl Study	80	34	0	Egyptian	Lifelong Premature Ejaculation	Paroxetine	Not Available	5-HTTLPR	Improvement in premature ejaculation		No statistically significant association between genotype and study outcome	11
Secher *et al.* (2009) [65]	19474754	Prospective Naturalistic Study	165	30-56	67	Danish Origin	Single Depressive Episode	Various Antidepressants	Not Available	5-HTTLPR	Weight Gain	UKU	No statistically significant	13

													association between genotype and study outcome	
Serretti *et al.* (2001) [66]	11526473	Prospective PlaceboControlled Study	217	52.1	66	Not Available	MDD and/or Bipolar Disorder	Fluvoxamine	Pindolol, Flurazepam, Lithium	5-HTTLPR	Clinical Outcomes	Hamilton Rating Scale for Depression	5-HTTLPR polymorphism was not independently assessed from A218C tryptophan hydroxylase (TPH), therefore no relevant conclusion was made	17
Serretti *et al.* (2004) [67]	15274037	Retrospecti ve Cohort Study	221	50.6	66	Not Available	MDD and/or Bipolar Disorder	Fluvoxamine, Paroxetine	Flurazepam, Lithium	5-HTTLPR	Clinical Outcomes	HAMD	Carriers of the SS genotype showed a selective and slower improvement of depressive “core” and somatic anxiety symptoms when taking SSRI’s	17
Serretti *et al.* (2007) [68]	17157919	Retrospecti ve Cohort Study	281	48.6	68	Not Available	MDD, Bipolar Disorder Type I or Bipolar Disorder Type II	Fluvoxamine, Paroxetine, Sertraline	Flurazepam, Lithium	5-HTTLPR	Clinical Outcomes	HAMD	When participants with the SL and LL genotypes were pooled together and compared to the SS genotype, they showed a significantly better outcome	18
Silva *et al.* (2010) [69]	20010449	Prospective Cohort Study	49	30	73	Not Available	PD	Fluoxetine	Not Available	5-HTTLPR	Clinical Outcomes	OAS-M	Participants with the LL genotype had a significantly better response to Fluoxetine than participants carrying the S allele	11
Smits *et al.* (2007) [70]	17414739	Retrospecti ve Cohort Study	214	48.48	70	Caucasian (Netherland s Population)	MDD	Paroxetine, Fluoxetine, Fluvoxamine, Sertraline, Citalopram	Not Available	5-HTTLPR, 5-HTTVNTR	Clinical Outcomes and Adverse Events	6 Main classes of adverse events	Participants with the SS or SL genotype appeared to have an increased risk for adverse events (especially general adverse events such as dermatologic reactions, weight change and fatigue)	14

Staeker *et al.* (2014) [71]	24192302	Prospective Naturalistic Study	273	Range: 18-89	64	Caucasian	Psychiatric disorders that require antidepressant s	Citalopram, Fluoxetine, Paroxetine, Sertraline, Duloxetine, Venlafaxine, Reboxetine, Amitriptyline, Clomipramine, Imipramine, Trimipramine Mirtazapine	Doxepin, Benzodiazepi nes Zopiclone Zolpidem Clomethiazole Antihypertens ive drugs: ACE inhibitors Beta-blockers Angiotensin II receptor antagonists Calciumchannel blockers Diuretics Cardiac glycosides Thyroid drugs: Lthyroxine Kalium iodide Antidiabetic drugs: Insulin Metformin Repaglinide Glimepiride Pioglitazone	5-HTTLPR, 5-HTTLPR/rs 25531, 5-HTTVNTR	Clinical Outcomes and Adverse Events	PD-S, CGI, DOTES	No statistically significant association between genotype and study outcome	13
Stein *et al.* (2006) [72]	16525856	Prospective Cohort Study	32	37.6	28	Caucasian (77%), Asian (8%), Other (8%), Black (6%), Indian/Alas kan Native/ First Nations (1%)	Social Anxiety Disorder	Paroxetine, Fluvoxamine	Not Available	5-HTTLPR	Clinical Outcomes	CGI-C	No statistically significant association between genotype and study outcome, however a statistical trend was found with LL carriers being more likely to respond than SS carriers	11
Stein *et al.* (2014) [73]	24154666	Prospective Cohort Study	346	35	34	Caucasian (77%), Asian (8%), Black (6%), Other (8%), American Indian/Alas ka Native (1%)	Social Anxiety Disorder	Sertraline	Not Available	5-HTTLPR	Clinical Outcomes	LSAS	No statistically significant association between genotype and study outcome	13

Strohmaier *et al.* (2011) [74]	21388237	Prospective Cohort Study	494	42	64	Not Available	MDD	Escitalopram, Nortriptyline	Hypnotics	5-HTTLPR	Clinical Outcomes, Sexual Dysfunction	MADRS, ASEC	No statistically significant association between genotype and study outcome	12
Sugie *et al.* (2005) [75]	16119478	Prospective PlaceboControlled Study	19	2-7	21	Japanese Population	ASD	Fluvoxamine	Not Available	5-HTTLPR	Clinical Outcomes, Side Effects	CGI	Fluvoxamine tended to be more effective in patients with the LL/LS genotype than those with the SS genotype. However, with respect to language use, a significant effectiveness was noted in the S allele	12
Takahashi *et al.* (2002) [76]	12208565	Prospective Cohort Study	54	51.2	59	Japanese	MDD	Fluvoxamine	Brotizolam	5-HTTLPR, 5-HTTVNTR	Clinical Outcomes, Nausea	MADRS, UKU	No statistically significant association between genotype and study outcome	12
Tomita *et al.* (2014) [77]	24858363	Prospective Cohort Study	51	46.3	69	Japanese	MDD	Paroxetine	Diazepam, Brotizolam, Sennoside	5-HTTLPR	Clinical Outcomes, Plasma Concentration	MADRS, UKU, Liquid Chromatograp hy	In the SS group, the paroxetine plasma concentration was significantly negatively correlated with improvement in MADRS at week 6 while the SL and LL group was significantly positively correlated. In the SS group, only the paroxetine plasma concentration showed a significant association with improvement in MADRS at week 6. However, in the SL and LL group, the paroxetine plasma concentration was not significantly associated with improvement in MADRS at week 6	14

Wilkie *et al.* (2009) [78]	18253134	Prospective Cohort Study	166	43.42	69	Caucasian	Unipolar Depression	Paroxetine, Citalopram, Imipramine, Lofepramine, Phenelzine	Not Available	5-HTTLPR, 5-HTTVNTR	Clinical Outcomes	HAMD	Participants with the S allele were significantly associated with both remission and response	13
Won *et al.* (2012) [79]	23095326	Prospective Cohort Study	115	46	86	Korean	MDD	Escitalopram	Lorazepam	5-HTTLPR	Clinical Outcomes	UKU, HAMD, CGI	The therapeutic response was better in S allele carriers than participants with the LL genotype	14
Wong *et al.* (2008) [80]	18311688	Prospective CaseControl Study	23	Mean betwee n 32-36	0	Not Available	Alcohol Dependency	Citalopram	Not Available	5-HTTLPR	Clinical Outcomes, ACTH Blood Levels	VAS, SSS	No statistically significant association between genotype and study outcome	12
Yevtushenk o *et al.* (2010) [81]	19800133	Prospective Cohort Study	102	36.9	76	Caucasian	Panic Disorder	Sertraline, Paroxetine	Not Available	5-HTTLPR	Frequency of Panic Attacks	HADS, CGI	No statistically significant association between genotype and study outcome	14
Zanardi *et al.* (2001) [82]	11543734	Prospective Cohort Study	155	51.97	70	Not Available	MDD and/or Bipolar Disorder	Fluvoxamine	Pindolol, Flurazepam, Lithium	5-HTTLPR	Clinical Outcomes	HAMD-21	The S allele was significantly associated with poorer response to fluvoxamine treatment	13

*Abbreviations used: ABC-CV, Aberrant Behavioral Checklist-Community Version; ASEC, Antidepressant Side-Effect Checklist; ASD, Autism Spectrum Disorder; BAI, Beck Anxiety Inventory; BDI, Beck Depression Inventory; BPRS, Brief Psychiatric Rating Scale; CAPS, Clinician-Administered PTSD Scale; CDRS-R, Children's Depression Rating ScaleRevised; CGI-I, Clinical Global Impression Scale- Improvement; CGI-S, Clinical Global Impression Severity; CIRS-G, Cumulative Illness Rating Scale adapted for Geriatrics; CSFQ,
Changes in Sexual Function Questionnaire; CTTH, Chronic Tension-Type Headache; DOTES, Dosage Record and Treatment Emergent Symptoms; FTND, Fagerstrom Test of Nicotine Dependence; FQ, Fear Questionnaire; GGTP, Concentration of F-glutamyl-transpeptidase; HADS, Hospital Anxiety and Depression Subscales; HAM-A, The Hamilton Anxiety Rating Scale; HAM-D, Hamilton Depression Rating Scale; IES[R], Impact of Event Scale-Revised; IVR, Interactive Voice Response Technology; K-SADS-PL, Hebrew version of the Schedule for Affective Disorders and Schizophrenia for School-Age Children-Present and Lifetime Version; LUNSERS, Liverpool University Neuroleptic Side Effect Rating Scale; MADRS, Montgomery-Asberg Depression Rating Scale; MMSE, Mini Mental Status Examination; MoCA, Montreal Cognitive Assessment; MSEL, Mullen Scales of Early Learning; NIHSS, National Institute of Health Stroke Scale; OAS-M, Overt Aggression Scale Modified; PANSS, Positive and Negative Syndrome Scale; PASS, Panic Associated Symptom Scale; PD-S, Paranoid Depression Scale; PDSS, Panic Disorder Severity Scale; PFA, Platelet Function Analyser; PSWQ, Penn State Worry Questionnaire; QIDS-C, 16-item Quick Inventory of Depressive Symptomatology Clinician Rated; RBS-R, Repetitive Behavior Scale-Revised; SCARED, Screen for the Child Anxiety Related Emotional Disorders; SIP, Short Index of Problems; SPM, Sensory Processing Measures; SSS, Serotonin Syndrome Scale; TLFB, Timeline Follow-Back; UKU, Udvalg for Kliniske Undersogelser; VAS, Visual Analog Scale; YMRS, Young Mania Rating Scale.

Table S2. Summary of Quality Score of Included Studies.

Study Author et al.	1. Choosing the genes/SNPs to genotype				2. Sample Size			3. Study Design	4. Reliability of Genotypes					5. Missing Genotype Data						6. Population Stratification		7. HardyWeinberg Equilibrium (HWE)			8. Choice and Definition of Outcomes			Quality Score
	Was a literature review undertaken and the findings summarized?	*Are reasons given for choosing the genes and SNPs genotyped?*	*Is method to adjust for multiple testing described?*	*Are precise pvalues provided for all associations?*	*What is the sample size?*	*Are details given of how the sample size was calculated?*	*Are details given of the a priori power to detect effect sizes of varying degrees?*	*What is the study design?*	*Is the genotyping procedure described?*	*Are the primers described?*	*Were quality control methods used and described?*	*Are any genotype frequencies previously reported quoted?*	*Were genotyping personnel blinded to outcome status?*	*Is extent of missing data summarized?*	*Where extent is summarized are reasons for missing data given?*	*Are checks for missingness at random reported?*	*Is missing genotype data imputed?*	*Does paper quote number of patients contributing to each analysis?*	*If paper does quote number of patients contributing to analyses does this agree to sample size?*	*Are tests undertaken for cryptic population stratification?*	*Is cryptic population stratification adjusted for in the analyses?*	*Was HWE tested?*	*Where test undertaken, are SNPs deviating from HWE highlighted?*	*Where test undertaken, and some SNPs found to deviate, are deviating SNPs excluded from further analysis?*	*Does the paper clearly define all outcomes investigated?*	*Is justification provided for the choice of outcomes?*	*Are results shown for all outcomes mentioned?*	
Abdelmalik et al. (2009)	YES	YES	NO	YES	19	NO	NO	PROSPECTIVE COHORT STUDY	YES	YES	NO	YES	NO	NO	N/A	N/A	N/A	YES	YES	NO	NO	NO	N/A	N/A	YES	YES	YES	11
Akcali et al. (2008)	YES	YES	NO	YES	264	NO	NO	PROSPECTIVE CASE CONTROL STUDY	YES	NO	YES	NO	YES	NO	N/A	N/A	N/A	YES	YES	NO	NO	YES	NO	N/A	YES	YES	YES	12
AlOlaby et al. (2017)	YES	YES	NO	YES	51	NO	NO	PROSPECTIVE PLACEBO-CONTROLLED STUDY	YES	YES	NO	NO	NO	YES	YES	NO	NO	YES	YES	NO	NO	NO	N/A	N/A	YES	YES	YES	12
Basu et al. (2015)	YES	YES	NO	YES	55	NO	NO	PROSPECTIVE COHORT STUDY	YES	NO	YES	NO	YES	YES	YES	NO	NO	YES	YES	NO	NO	YES	NO	N/A	YES	YES	YES	14
Baumer et al. (2006)	YES	YES	YES	YES	52	NO	NO	RETROSPECTIVE CASE CONTROL STUDY	YES	YES	YES	NO	YES	YES	YES	NO	NO	YES	YES	NO	NO	YES	YES	NO	YES	YES	YES	17
Billett et al. (1997)	YES	YES	NO	YES	144	NO	YES	CASE CONTROL STUDY	YES	NO	NO	NO	NO	NO	N/A	N/A	N/A	YES	YES	NO	NO	YES	NO	N/A	YES	YES	YES	11
Bishop et al. (2009)	YES	YES	NO	YES	115	YES	YES	PROSPECTIVE COHORT STUDY	YES	YES	NO	NO	NO	NO	N/A	N/A	N/A	YES	YES	NO	NO	YES	NO	N/A	YES	YES	YES	13
Bloch et al. 2010	YES	YES	NO	YES	61	NO	NO	PROSPECTIVE PLACEBO CONTROLLED STUDY	YES	YES	NO	NO	NO	YES	YES	NO	NO	YES	YES	NO	NO	NO	N/A	N/A	YES	YES	YES	12
de Aguiar Ferreira et al. (2009)	YES	YES	NO	YES	112	NO	NO	RETROSPECTIVE CASE CONTROL STUDY	YES	YES	NO	NO	NO	NO	N/A	N/A	N/A	YES	YES	NO	NO	YES	NO	N/A	YES	YES	YES	11
Denys et al. (2007)	YES	YES	YES	YES	91	NO	NO	PROSPECTIVE PARALLEL-GROUP STUDY	YES	YES	NO	NO	NO	YES	YES	NO	NO	YES	YES	NO	NO	NO	N/A	N/A	YES	YES	YES	13
Di Bella et al. (2002)	YES	YES	NO	YES	372	NO	YES	PROSPECTIVE CASE CONTROL STUDY	YES	YES	YES	NO	YES	YES	YES	NO	NO	YES	YES	NO	NO	YES	NO	N/A	YES	YES	YES	16
Dombrovski et al. (2010)	YES	YES	NO	YES	92	NO	NO	PROSPECTIVE COHORT STUDY	YES	YES	NO	NO	NO	YES	YES	NO	NO	YES	YES	NO	NO	YES	NO	N/A	YES	YES	YES	13
Durham et al. (2004)	YES	YES	NO	YES	217	NO	NO	PROSPECTIVE PLACEBO-CONTROLLED STUDY	YES	NO	NO	NO	NO	YES	NO	NO	NO	YES	YES	NO	NO	YES	NO	N/A	YES	YES	YES	11
Frye et al. (2015)	YES	YES	NO	YES	295	NO	NO	RETROSPECTIVE CASE CONTROL STUDY	YES	NO	NO	NO	NO	NO	N/A	N/A	N/A	YES	YES	NO	NO	NO	N/A	N/A	YES	YES	YES	9
Garfield et al. (2014)	YES	YES	NO	YES	177	NO	YES	PROSPECTIVE PLACEBO CONTROLLED STUDY	YES	NO	NO	NO	NO	YES	YES	NO	NO	YES	YES	NO	NO	YES	NO	N/A	YES	YES	YES	13
Gressier et al. (2014)	YES	YES	NO	YES	103	NO	NO	NATURALISTIC PROSPECTIVE STUDY	YES	NO	NO	NO	NO	NO	N/A	N/A	N/A	YES	YES	NO	NO	YES	NO	N/A	YES	YES	YES	10
Han et al. (2008)	YES	YES	NO	YES	225	NO	NO	PROSPECTIVE COHORT STUDY	YES	YES	NO	NO	NO	NO	N/A	N/A	N/A	YES	YES	NO	NO	YES	NO	N/A	YES	YES	YES	11
Higuchi et al. (2009)	YES	YES	YES	YES	80	NO	NO	PROSPECTIVE COHORT STUDY	YES	NO	NO	NO	NO	YES	YES	NO	NO	YES	YES	NO	NO	YES	YES	YES	YES	YES	YES	15
Hougardy et al. (2008)	YES	YES	NO	NO	43	NO	NO	NATURALISTIC PROSPECTIVE STUDY	YES	NO	NO	YES	NO	YES	YES	NO	NO	YES	YES	NO	NO	NO	N/A	N/A	YES	YES	YES	11
Hu et al. (2007)	YES	YES	NO	YES	2626	NO	NO	PROSPECTIVE CASE CONTROL STUDY	YES	NO	NO	NO	NO	YES	YES	NO	NO	YES	YES	NO	NO	NO	N/A	N/A	YES	YES	YES	11
Huezo-Diaz et al. (2009)	YES	YES	NO	YES	810	YES	YES	PROSPECTIVE COHORT STUDY	YES	NO	NO	NO	NO	YES	NO	NO	NO	YES	YES	NO	NO	YES	YES	NO	YES	YES	YES	14
Ishiguro et al. (2011)	YES	YES	NO	YES	65	NO	NO	PROSPECTIVE COHORT STUDY	YES	YES	NO	NO	NO	YES	YES	NO	NO	YES	YES	NO	NO	NO	N/A	N/A	YES	YES	YES	12
Joyce at al. (2005)	YES	NO	NO	YES	195	NO	NO	PROSPECTIVE COHORT STUDY	YES	YES	NO	NO	NO	YES	NO	NO	NO	YES	YES	NO	NO	NO	N/A	N/A	YES	YES	YES	10
Kato et al. (2006)	YES	YES	NO	YES	100	NO	NO	PROSPECTIVE COHORT STUDY	YES	NO	NO	NO	NO	YES	YES	NO	YES	YES	YES	NO	NO	YES	NO	N/A	YES	YES	YES	13
Kenna et al. (2009)	YES	YES	NO	YES	15	NO	NO	PROSPECTIVE COHORT STUDY	YES	YES	NO	NO	NO	YES	YES	NO	NO	YES	YES	NO	NO	NO	N/A	N/A	YES	YES	YES	12
Kenna et al. (2014)	YES	YES	NO	YES	49	NO	NO	PROSPECTIVE PLACEBO CONTROLLED STUDY	YES	YES	YES	NO	YES	YES	YES	NO	NO	YES	YES	NO	NO	YES	NO	N/A	YES	YES	YES	15
Kranzler et al. (2011)	YES	YES	NO	YES	134	NO	NO	PROSPECTIVE PLACEBO CONTROLLED STUDY	YES	YES	NO	NO	NO	YES	NO	NO	NO	YES	YES	NO	NO	YES	NO	N/A	YES	YES	YES	12
Kranzler et al. (2012)	YES	YES	NO	YES	134	NO	NO	PROSPECTIVE CASE CONTROL STUDY	YES	NO	NO	NO	NO	YES	NO	NO	NO	YES	YES	NO	NO	NO	N/A	N/A	YES	YES	YES	10
Kranzler et al. (2013)	YES	YES	NO	YES	134	NO	NO	PROSPECTIVE PLACEBO CONTROLLED STUDY	YES	YES	NO	NO	NO	YES	NO	NO	NO	YES	YES	NO	NO	YES	NO	N/A	YES	YES	YES	12
Kronenberg et al. (2007)	YES	YES	NO	YES	74	NO	NO	PROSPECTIVE COHORT STUDY	YES	NO	NO	NO	NO	YES	YES	NO	NO	YES	YES	NO	NO	YES	NO	N/A	YES	YES	YES	12
Lanctôt et al. (2010)	YES	YES	NO	YES	90	NO	NO	PROSPECTIVE COHORT STUDY	YES	NO	NO	NO	NO	YES	YES	NO	NO	YES	YES	NO	NO	YES	NO	N/A	YES	YES	YES	12
Lee et al. (2010)	YES	YES	NO	YES	84	NO	NO	PROSPECTIVE COHORT STUDY	YES	YES	NO	NO	NO	YES	YES	NO	NO	YES	YES	NO	NO	YES	NO	N/A	YES	YES	YES	13
Lee et al. (2018)	YES	YES	NO	YES	301	NO	NO	PROSPECTIVE PLACEBO CONTROLLED STUDY	YES	YES	NO	NO	NO	YES	NO	NO	NO	YES	YES	NO	NO	YES	NO	N/A	YES	YES	YES	12
Lenze et al. 2010	YES	YES	NO	YES	150	NO	NO	PROSPECTIVE PLACEBO-CONTROLLED STUDY	YES	NO	NO	YES	NO	YES	YES	NO	NO	YES	YES	NO	NO	YES	NO	N/A	YES	YES	YES	13
Liu et al. (2018)	YES	YES	YES	YES	244	NO	NO	PROSPECTIVE CASE CONTROL STUDY	YES	YES	NO	NO	NO	YES	YES	NO	NO	YES	YES	NO	NO	YES	NO	N/A	YES	YES	YES	14
Lohoff et al. (2013)	YES	YES	NO	YES	112	NO	NO	PROSPECTIVE COHORT STUDY	YES	NO	YES	YES	NO	NO	N/A	N/A	N/A	YES	YES	NO	NO	YES	NO	N/A	YES	YES	YES	12
Mandelli et al. (2009)	YES	YES	NO	YES	86	NO	NO	PROSPECTIVE COHORT STUDY	YES	YES	NO	YES	NO	YES	NO	NO	NO	NO	YES	NO	NO	YES	YES	NO	YES	YES	YES	13
Maron et al. (2009)	YES	YES	YES	YES	135	NO	NO	PROSPECTIVE PLACEBO-CONTROLLED STUDY	YES	YES	NO	NO	NO	YES	YES	NO	NO	YES	YES	NO	NO	YES	NO	N/A	YES	YES	YES	14
Masoliver et al. (2006)	YES	YES	NO	YES	288	NO	NO	PROSPECTIVE CASE CONTROL STUDY	YES	YES	NO	NO	NO	NO	N/A	N/A	N/A	YES	YES	NO	NO	YES	NO	N/A	YES	YES	YES	11
Miguita et al. (2011)	YES	YES	NO	YES	41	NO	NO	PROSPECTIVE COHORT STUDY	YES	YES	YES	NO	NO	NO	N/A	N/A	N/A	YES	YES	NO	NO	YES	NO	N/A	YES	YES	YES	12
Monteleone et al. (2005)	YES	YES	NO	YES	47	NO	NO	PROSPECTIVE NATURALISTIC STUDY	YES	YES	NO	NO	NO	NO	N/A	N/A	N/A	YES	YES	NO	NO	YES	NO	N/A	YES	YES	YES	11
Murata et al. (2010)	YES	YES	YES	YES	56	NO	NO	PROSPECTIVE COHORT STUDY	YES	YES	NO	NO	NO	YES	YES	NO	NO	YES	YES	NO	NO	YES	NO	N/A	YES	YES	YES	14
Murphy et al. (2004)	YES	YES	NO	YES	246	NO	NO	PROSPECTIVE COHORT STUDY	YES	NO	NO	NO	NO	YES	YES	NO	NO	YES	YES	NO	NO	YES	NO	N/A	YES	YES	YES	12
Mushtaq et al. (2012)	YES	YES	YES	YES	226	YES	YES	PROSPECTIVE COHORT STUDY	YES	YES	NO	NO	NO	YES	YES	NO	NO	YES	YES	NO	NO	NO	N/A	N/A	YES	YES	YES	15
Najjar et al. (2015)	YES	YES	NO	YES	44	NO	NO	PROSPECTIVE COHORT STUDY	YES	YES	NO	NO	NO	YES	NO	NO	NO	YES	YES	NO	NO	YES	NO	N/A	YES	YES	YES	12
Ng et al. (2006)	YES	YES	NO	YES	45	NO	NO	PROSPECTIVE COHORT STUDY	YES	YES	NO	YES	NO	NO	N/A	N/A	N/A	YES	YES	NO	NO	NO	N/A	N/A	YES	YES	YES	11
Ng et al. (2013)	YES	YES	YES	YES	106	NO	NO	PROSPECTIVE COHORT STUDY	YES	NO	NO	NO	NO	NO	N/A	N/A	N/A	YES	YES	NO	NO	YES	NO	N/A	YES	YES	YES	11
Ng et al. (2016)	YES	YES	NO	YES	35	YES	YES	PROSPECTIVE COHORT STUDY	YES	NO	YES	NO	NO	NO	N/A	N/A	N/A	YES	YES	NO	NO	YES	NO	N/A	YES	YES	YES	13
Owley et al. (2010)	YES	YES	NO	YES	58	NO	NO	PROSPECTIVE COHORT STUDY	YES	YES	NO	NO	NO	YES	YES	NO	NO	YES	YES	NO	NO	YES	NO	N/A	YES	YES	YES	13
Ozbek et al. (2014)	YES	YES	NO	YES	69	NO	NO	PROSPECTIVE COHORT STUDY	YES	YES	YES	NO	NO	NO	N/A	N/A	N/A	YES	YES	NO	NO	YES	NO	N/A	YES	YES	YES	12
Park et al. (2016)	YES	YES	NO	YES	24	NO	NO	PROSPECTIVE COHORT STUDY	YES	YES	NO	NO	NO	NO	N/A	N/A	N/A	YES	YES	NO	NO	NO	N/A	N/A	YES	YES	YES	10
Perlis et al. (2003)	YES	YES	NO	YES	36	NO	NO	PROSPECTIVE COHORT STUDY	YES	YES	NO	NO	NO	YES	YES	NO	NO	YES	YES	NO	NO	NO	N/A	N/A	YES	YES	YES	12
Perna et al. 2005	YES	YES	YES	NO	92	NO	NO	PROSPECTIVE COHORT STUDY	YES	YES	YES	NO	YES	NO	N/A	N/A	N/A	YES	YES	NO	NO	YES	NO	N/A	YES	YES	YES	13
Peters et al. (2016)	YES	YES	NO	YES	175	NO	NO	RETROSPECTIVE PLACEBO-CONTROLLED STUDY	YES	YES	NO	NO	NO	YES	YES	NO	NO	YES	YES	NO	NO	NO	N/A	N/A	YES	YES	YES	12
Popp et al. (2006)	YES	YES	NO	YES	109	NO	NO	RETROSPECTIVE COHORT STUDY	YES	YES	NO	NO	NO	YES	YES	NO	NO	YES	YES	NO	NO	YES	NO	N/A	YES	YES	YES	13
Putzhammer et al. (2005)	YES	YES	NO	YES	62	NO	NO	PROSPECTIVE COHORT STUDY	YES	YES	NO	NO	NO	YES	YES	NO	NO	YES	YES	NO	NO	YES	NO	N/A	YES	YES	YES	13
Quaak et al. (2012)	YES	YES	NO	YES	214	NO	NO	PROSPECTIVE PLACEBO-CONTROLLED STUDY	YES	YES	NO	NO	NO	NO	N/A	N/A	N/A	YES	YES	NO	NO	YES	NO	N/A	YES	YES	YES	11
Rahikainen et al. (2017)	YES	YES	NO	YES	349	NO	NO	RETROSPECTIVE MATCHED CASE STUDY	YES	YES	NO	NO	NO	NO	N/A	N/A	N/A	YES	YES	NO	NO	YES	YES	NO	YES	YES	YES	12
Rotberg et al. (2013)	YES	YES	YES	YES	83	NO	NO	PROSPECTIVE/RETROSPECTIVE COHORT STUDY	YES	YES	YES	NO	YES	YES	YES	NO	NO	YES	YES	NO	NO	YES	NO	N/A	YES	YES	YES	16
Rousseva et al. (2003)	YES	YES	NO	YES	305	YES	YES	CASE CONTROL STUDY	YES	YES	NO	NO	NO	NO	N/A	N/A	N/A	YES	YES	NO	NO	YES	NO	N/A	YES	YES	YES	13
Saeki et al. (2009)	YES	YES	NO	YES	27	NO	NO	PROSPECTIVE COHORT STUDY	YES	YES	NO	NO	NO	YES	YES	NO	NO	YES	YES	NO	NO	NO	N/A	N/A	YES	YES	YES	12
Sahraian et al. (2013)	YES	YES	NO	YES	104	NO	NO	PROSPECTIVE COHORT STUDY	YES	YES	YES	NO	YES	YES	YES	NO	NO	YES	YES	NO	NO	NO	N/A	N/A	YES	YES	YES	14
Saiz-Rodríguez et al. (2018)	YES	YES	NO	YES	46	NO	NO	PROSPECTIVE COHORT STUDY	YES	NO	NO	NO	NO	YES				YES	YES	NO	NO	YES	NO	N/A	YES	YES	YES	11
Salem et al. (2017)	YES	YES	NO	YES	80	NO	NO	PROSPECTIVE CASE CONTROL STUDY	YES	YES	NO	NO	NO	NO	N/A	N/A	N/A	YES	YES	NO	NO	YES	NO	N/A	YES	YES	YES	11
Secher et al. (2009)	YES	YES	NO	YES	165	NO	NO	PROSPECTIVE NATURALISTIC STUDY	YES	YES	NO	NO	NO	YES	YES	NO	NO	YES	YES	NO	NO	YES	NO	N/A	YES	YES	YES	13
Serretti et al. (2001)	YES	YES	NO	YES	217	YES	YES	PROSPECTIVE PLACEBO-CONTROLLED STUDY	YES	YES	YES	NO	YES	YES	YES	NO	NO	YES	YES	NO	NO	YES	NO	N/A	YES	YES	YES	17
Serretti et al. (2004)	YES	YES	NO	YES	221	YES	YES	PROSPECTIVE COHORT STUDY	YES	YES	YES	NO	YES	YES	YES	NO	NO	YES	YES	NO	NO	YES	NO	N/A	YES	YES	YES	17
Serretti et al. (2007)	YES	YES	YES	YES	281	YES	YES	PRSPECTIVE COHORT STUDY	YES	YES	YES	NO	YES	YES	YES	NO	NO	YES	YES	NO	NO	YES	NO	N/A	YES	YES	YES	18
Silva et al. (2010)	YES	YES	NO	YES	49	NO	NO	PROSPECTIVE COHORT STUDY	YES	NO	NO	NO	NO	YES	NO	NO	NO	YES	YES	NO	NO	YES	NO	N/A	YES	YES	YES	11
Smits et al. (2007)	YES	YES	NO	YES	214	NO	NO	PROSPECTIVE COHORT STUDY	YES	YES	YES	NO	YES	YES	YES	NO	NO	YES	YES	NO	NO	YES	NO	N/A	YES	YES	YES	15
Staeker et al. (2014)	YES	YES	NO	YES	273	NO	NO	PROSPECTIVE NATURALISTIC STUDY	YES	NO	NO	YES	NO	YES	YES	NO	NO	YES	YES	NO	NO	YES	NO	N/A	YES	YES	YES	13
Stein et al. (2006)	YES	YES	NO	YES	32	NO	NO	PROSPECTIVE COHORT STUDY	YES	YES	NO	NO	NO	NO	N/A	N/A	N/A	YES	YES	NO	NO	YES	NO	N/A	YES	YES	YES	11
Stein et al. (2014)	YES	YES	YES	YES	346	NO	NO	PROSPECTIVE COHORT STUDY	YES	YES	NO	NO	NO	YES	YES	NO	NO	YES	YES	NO	NO	NO	N/A	N/A	YES	YES	YES	13
Strohmaier et al. (2011)	YES	YES	NO	YES	494	NO	NO	PROSPECTIVE COHORT STUDY	YES	NO	NO	YES	NO	YES	NO	NO	NO	YES	YES	NO	NO	YES	NO	N/A	YES	YES	YES	12
Sugie et al. (2005)	YES	YES	NO	YES	19	NO	NO	PROSPECTIVE PLACEBO-CONTROLLED STUDY	YES	YES	NO	NO	NO	YES	YES	NO	NO	YES	YES	NO	NO	NO	N/A	N/A	YES	YES	YES	12
Takahashi et al. (2002)	YES	YES	NO	YES	54	NO	NO	PROSPECTIVE COHORT STUDY	YES	YES	NO	NO	NO	YES	YES	NO	NO	YES	YES	NO	NO	NO	N/A	N/A	YES	YES	YES	12
Tomita et al. (2014)	YES	YES	NO	YES	51	NO	NO	PROSPECTIVE COHORT STUDY	YES	YES	NO	YES	NO	YES	YES	NO	NO	YES	YES	NO	NO	YES	NO	N/A	YES	YES	YES	14
Wilkie et al. (2009)	YES	YES	NO	YES	166	NO	NO	PROSPECTIVE COHORT STUDY	YES	YES	NO	YES	NO	YES	YES	NO	NO	YES	YES	NO	NO	NO	N/A	N/A	YES	YES	YES	13
Won et al. (2012)	YES	YES	NO	YES	115	NO	NO	PROSPECTIVE COHORT STUDY	YES	YES	NO	YES	NO	YES	YES	NO	NO	YES	YES	NO	NO	YES	NO	N/A	YES	YES	YES	14
Wong et al. (2008)	YES	YES	YES	YES	23	NO	NO	PROSPECTIVE CASE CONTROL STUDY	YES	YES	NO	NO	NO	NO	N/A	N/A	N/A	YES	YES	NO	NO	YES	NO	N/A	YES	YES	YES	12
Yevtushenko et al. (2010)	YES	YES	NO	YES	102	NO	NO	PROSPECTIVE COHORT STUDY	YES	YES	NO	YES	NO	YES	YES	NO	NO	YES	YES	NO	NO	YES	NO	N/A	YES	YES	YES	14

Zanardi et al. (2001)	YES	YES	NO	YES	155	NO	YES	PROSPECTIVE COHORT STUDY	YES	YES	NO	NO	NO	YES	YES	NO	NO	YES	YES	NO	NO	NO	N/A	N/A	YES	YES	YES	13

Supplementary Table S3. Reported prevalence of ADRs among studies in the tolerability metaanalysis

Study	Subjects Reporting ADR/Total Subjects	Prevalence (%)
Basu 2015 [4]	34/55	61.8
Bishop 2009 [7]	85/108	78.7
Higuchi 2009a [18]	9/80	11.3
Higuchi 2009b [18]	9/80	11.3
Hu 2007 [20]	169/1655	10.2
Ishiguro 2011 [22]	9/65	5.5
Murata 2010 [42]	20/56	35.7
Murphy 2004a [43]	18/123	78.3
Murphy 2004b [43]	30/121	24.8
Saeki 2009 [61]	5/27	18.5
Smits 2007 [70]	180/209	86.1
Takahasi 2002 [76]	16/54	29.6
Wilkie 2009 [78]	4/76	5.3

(A)

Study or Subgroup	LL/LS Events	LL/LS Total	SS Events	SS Total	Weight	Odds Ratio IV, Random, 95% CI
Basu 2015	12	17	22	38	7.7%	1.75 [0.51, 5.95]
Bishop 2009	31	89	8	19	9.2%	0.73 [0.27, 2.02]
Higuchi 2009a	4	29	5	51	6.7%	1.47 [0.36, 5.98]
Higuchi 2009b	7	30	2	50	5.5%	7.30 [1.41, 37.97]
Hu 2007	124	1352	45	303	14.1%	0.58 [0.40, 0.84]
Ishiguro 2011	5	26	4	39	6.6%	2.08 [0.50, 8.63]
Murata 2010	6	24	14	32	8.2%	0.43 [0.13, 1.36]
Murphy 2004a	16	92	2	31	6.1%	3.05 [0.66, 14.11]
Murphy 2004b	18	97	12	24	9.7%	0.23 [0.09, 0.59]
Saeki 2009	3	9	2	18	4.2%	4.00 [0.53, 30.16]
Smits 2007	137	158	43	51	10.2%	1.21 [0.50, 2.94]
Takahasi 2002	5	24	11	30	7.7%	0.45 [0.13, 1.56]
Wilkie 2009	2	55	2	21	4.2%	0.36 [0.05, 2.73]
**Total (95% CI)**		**2002**		**707**	**100.0%**	**0.95 [0.59, 1.55]**
Total events	370		172			

Heterogeneity: $Tau^2 = 0.40$; $Chi^2 = 29.00$, df = 12 (P = 0.004); $I^2 = 59\%$

Test for overall effect: Z = 0.19 (P = 0.85)

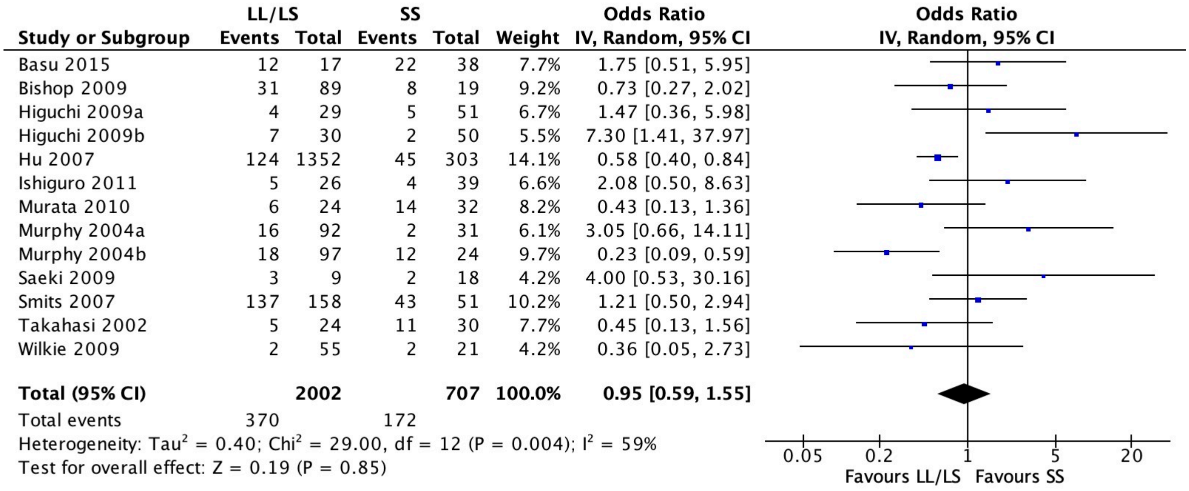

(B)

Study or Subgroup	Case Events	Case Total	Control Events	Control Total	Weight	Odds Ratio M-H, Random, 95% CI
Basu 2015	0	0	0	0		Not estimable
Bishop 2009	21	44	8	19	13.6%	1.26 [0.42, 3.72]
Higuchi 2009a	0	2	5	51	3.9%	1.69 [0.07, 39.95]
Higuchi 2009b	1	2	2	50	4.0%	24.00 [1.07, 539.11]
Hu 2007	50	620	45	303	18.9%	0.50 [0.33, 0.77]
Ishiguro 2011	0	3	4	39	4.0%	1.13 [0.05, 25.55]
Murata 2010	2	2	14	32	4.0%	6.38 [0.28, 143.49]
Murphy 2004a	10	38	2	31	9.8%	5.18 [1.04, 25.77]
Murphy 2004b	5	40	12	24	12.5%	0.14 [0.04, 0.49]
Saeki 2009	0	0	2	18		Not estimable
Smits 2007	59	72	43	51	14.7%	0.84 [0.32, 2.22]
Takahasi 2002	2	4	11	30	7.2%	1.73 [0.21, 14.05]
Wilkie 2009	2	24	2	21	7.4%	0.86 [0.11, 6.73]
**Total (95% CI)**		**851**		**669**	**100.0%**	**1.05 [0.52, 2.12]**
Total events	152		150			

Heterogeneity: $Tau^2 = 0.62$; $Chi^2 = 23.95$, df = 10 (P = 0.008); $I^2 = 58\%$

Test for overall effect: Z = 0.15 (P = 0.88)

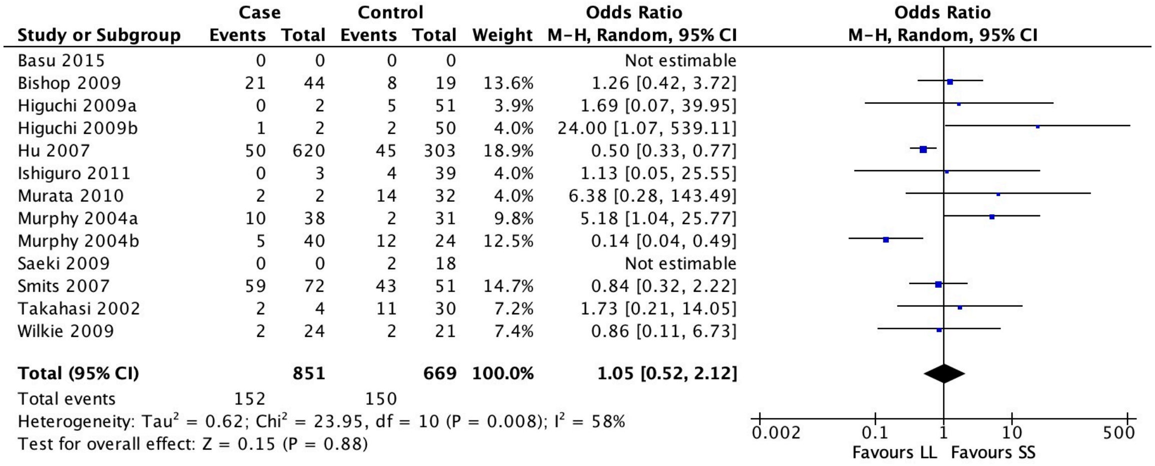

(C)

Study or Subgroup	Case Events	Case Total	Control Events	Control Total	Weight	Odds Ratio M-H, Fixed, 95% CI
Basu 2015	12	17	34	55	2.2%	1.48 [0.46, 4.81]
Bishop 2009	31	89	18	64	6.3%	1.37 [0.68, 2.74]
Higuchi 2009a	4	29	9	78	1.9%	1.23 [0.35, 4.34]
Higuchi 2009b	7	30	8	78	1.6%	2.66 [0.87, 8.15]
Hu 2007	124	1352	119	1035	56.1%	0.78 [0.60, 1.01]
Ishiguro 2011	5	26	9	62	2.0%	1.40 [0.42, 4.68]
Murata 2010	6	24	18	54	3.8%	0.67 [0.23, 1.97]
Murphy 2004a	16	92	8	85	3.1%	2.03 [0.82, 5.01]
Murphy 2004b	18	97	25	81	10.2%	0.51 [0.25, 1.02]
Saeki 2009	3	9	5	27	0.8%	2.20 [0.41, 11.95]
Smits 2007	137	158	121	137	7.9%	0.86 [0.43, 1.73]
Takahasi 2002	5	24	14	50	3.3%	0.68 [0.21, 2.16]
Wilkie 2009	2	55	2	52	0.9%	0.94 [0.13, 6.95]
**Total (95% CI)**		**2002**		**1858**	**100.0%**	**0.90 [0.75, 1.10]**
Total events	370		390			

Heterogeneity: $Chi^2 = 14.85$, df = 12 (P = 0.25); $I^2 = 19\%$

Test for overall effect: Z = 1.03 (P = 0.30)

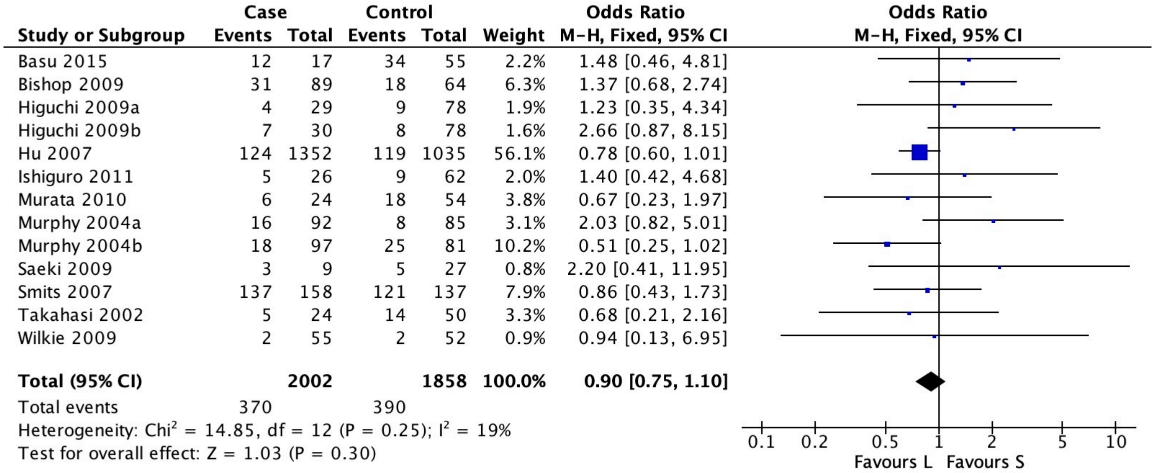

Figure S1. Forest plots of 5-HTTLPR polymorphisms and adverse drug reactions in all studies by genotype comparisons. (A) LL/LS vs. SS (B) LL vs. SS (C) L vs. S.

(A)

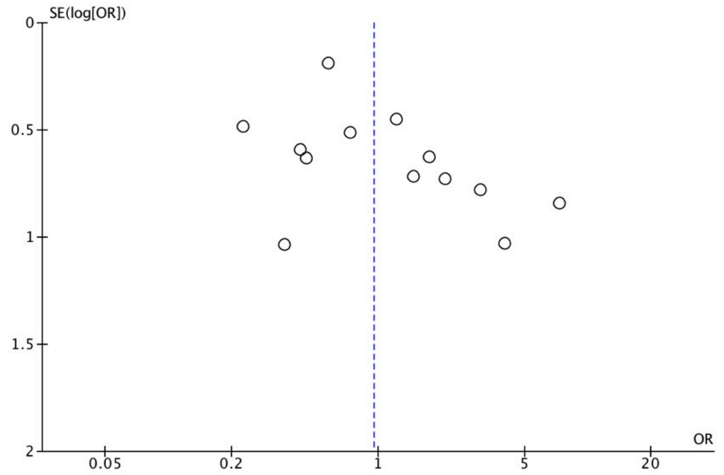

(B)

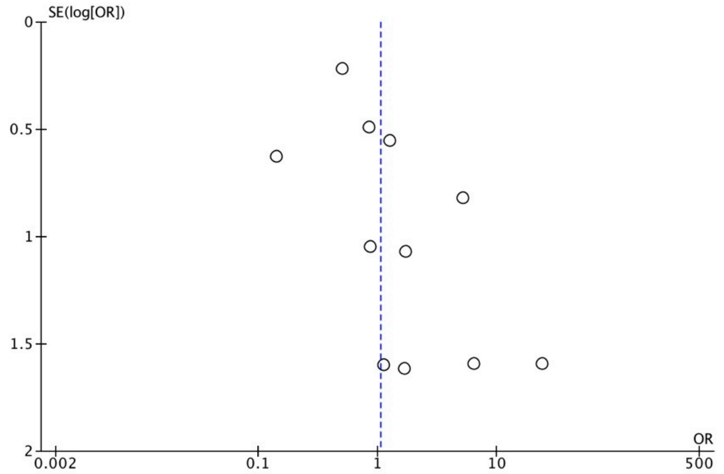

(C)

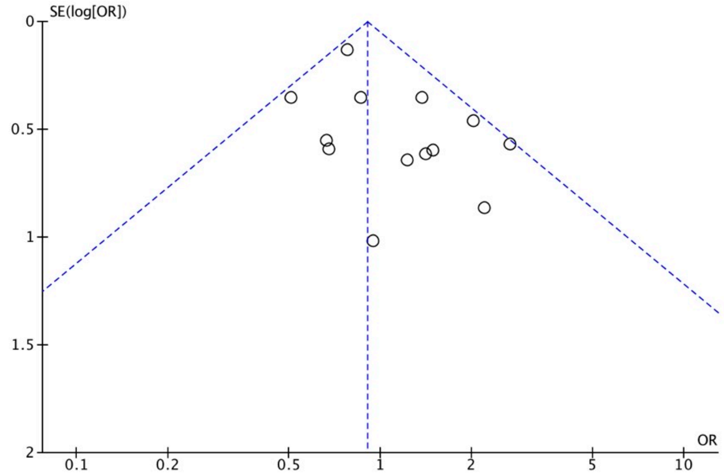

Figure S2. Funnel plots of 5-HTTLPR polymorphisms and adverse drug reactions in all studies by genotype comparisons. (A) LL/LS vs. SS (B) LL vs. SS (C) L vs. S

References:

1. Abdelmalik, N.; Ruhé, H.G.; Barwari, K.; van den Dool, E.J.; Meijers, J.C.; Middeldorp, S.; Büller, H.R.; Schene, A.H.; Kamphuisen, P.W.
Effect of the selective serotonin reuptake inhibitor paroxetine on platelet function is modified by a SLC6A4 serotonin transporter polymorphism. *J. Thromb. Haemost.* **2008**, *6*, 2168–2174, doi:10.1111/J.1538-7836.2008.03196.X.
2. Aylin, A.; Cengiz, T.; Emin, E.; Neriman, A.; Sacide, P. Serotonin transporter gene polymorphisms in patients with chronic tension-type headache: a preliminary study. *Neurol. India* **2008**, *56*, 156–160, doi:10.4103/0028-3886.41993.
3. AlOlaby, R.R.; Sweha, S.R.; Silva, M.; Durbin-Johnson, B.; Yrigollen, C.M.; Pretto, D.; Hagerman, R.J.; Tassone, F. Molecular biomarkers predictive of sertraline treatment response in young children with fragile X syndrome. *Brain Dev.* **2017**, *39*, 483–492, doi:10.1016/j.braindev.2017.01.012.
4. Basu, A.; Chadda, R. K.; Sood, M.; Kaur, H.; Kukreti, R. Association of serotonin transporter (SLC6A4) and receptor (5HTR1A, 5HTR2A) polymorphisms with response to treatment with escitalopram in patients with major depressive disorder: A preliminary study. *Indian J. Med. Res.* **2015**, *142*, 40–45, doi:10.4103/0971-5916.162094.
5. Baumer, F.M.; Howe, M.; Gallelli, K.; Simeonova, D.I.; Hallmayer, J.; Chang, K.D. A Pilot Study of AntidepressantInduced Mania in Pediatric Bipolar Disorder: Characteristics, Risk Factors, and the Serotonin Transporter Gene. *Biol. Psychiatry* **2006**, *60*, 1005–1012, doi:10.1016/j.biopsych.2006.06.010.
6. Billett, E.A.; Richter, M.A.; King, N., Heils, A.; Lesch, K.P.; Kennedy, J.L. Obsessive compulsive disorder, response to serotonin reuptake inhibitors and the serotonin transporter gene. *Mol. Psychiatry* **1997**, *2*, 403–406, doi:10.1038/SJ.MP.4000257.
7. Bishop, J.R.; Ellingrod, V.L.; Akroush, M.; Moline, J. The association of serotonin transporter genotypes and selective serotonin reuptake inhibitor (SSRI)-associated sexual side effects: possible relationship to oral contraceptives. *Hum. Psychopharmacol.* **2009**, *24*, 207–215, doi:10.1002/HUP.1006.
8. Bloch, B.; Reshef, A.; Cohen, T.; Tafla, A.; Gathas, S.; Israel, S.; Gritsenko, I.; Kremer, I.; Ebstein, R. P. Preliminary effects of bupropion and the promoter region (HTTLPR) serotonin transporter (SLC6A4) polymorphism on smoking behavior in schizophrenia. *Psychiatry Res.* **2010**, *175*, 38–42, doi:10.1016/J.PSYCHRES.2008.12.015.
9. Ferreira, A.; Neves, F.S.; da Rocha, F.F.; Silva, G.S.; Romano-Silva, M.A.; Miranda, D.M.; De Marco, L.; Correa, H. The role of 5-HTTLPR polymorphism in antidepressant-associated mania in bipolar disorder. *J. Affect. Disord.* **2009**, *112*, 267–272, doi:10.1016/J.JAD.2008.04.012.
10. Denys, D.; Van Nieuwerburgh, F.; Deforce, D.; Westenberg, H. G. Prediction of response to paroxetine and venlafaxine by serotonin-related genes in obsessive-compulsive disorder in a randomized, double-blind trial. *J. Clin. Psychiatry* **2007**, *68*, 747–753, doi:10.4088/JCP.V68N0512.
11. Di Bella, D.; Erzegovesi, S.; Cavallini, M. C.; Bellodi, L. Obsessive-Compulsive Disorder, 5-HTTLPR polymorphism and treatment response. *Pharmacogenomics J.* **2002**, *2*, 176–181, doi:10.1038/SJ.TPJ.6500090.
12. Dombrovski, A.Y.; Mulsant, B. H.; Ferrell, R. E.; Lotrich, F. E.; Rosen, J. I.; Wallace, M.; Houck, P. R.; Mazumdar, S.; Pollock, B. G. Serotonin transporter triallelic genotype and response to citalopram and risperidone in dementia with behavioral symptoms. *Int. Clin. Psychopharmacol.* **2010**, *25*, 37–45, doi:10.1097/YIC.0B013E328333EE10.
13. Durham, L.K.; Webb, S.M.; Milos, P.M.; Clary, C.M.; Seymour, A. B. The serotonin transporter polymorphism, 5HTTLPR, is associated with a faster response time to sertraline in an elderly population with major depressive disorder. *Psychopharmacology (Berl).* **2004**, *174*, 525–529, doi:10.1007/S00213-003-1562-3.
14. Frye, M.A.; McElroy, S.L.; Prieto, M.L.; Harper, K.L.; Walker, D.L.; Kung, S.; Chauhan, M.; Crow, S.; Sutor, B.; Galardy, C. W.; Veldic, M.; Palmer, B.A.; Geske, J.R.; Fuentes, M.; Cuellar-Barboza, A.B.; Seymour, L.R.; Mori, N.; Biernacka, J.M. Clinical risk factors and serotonin transporter gene variants associated with antidepressantinduced mania. *J. Clin. Psychiatry* **2015**, *76*, 174–180, doi:10.4088/JCP.14M09127.
15. Garfield, L.D.; Dixon, D.; Nowotny, P.; Lotrich, F.E.; Pollock, B.G.; Kristjansson, S.D.; Doré, P.M.; Lenze, E. J. Common selective serotonin reuptake inhibitor side effects in older adults associated with genetic polymorphisms in the serotonin transporter and receptors: data from a randomized controlled trial. *Am. J. Geriatr. Psychiatry* **2014**, *22*, 971–979, doi:10.1016/J.JAGP.2013.07.003.
16. Gressier, F.; Verstuyft, C.; Hardy, P.; Becquemont, L.; Corruble, E. Menopausal status could modulate the association between 5-HTTLPR and antidepressant efficacy in depressed women: a pilot study. *Arch. Womens. Ment. Health* **2014**, *17*, 569–573, doi:10.1007/S00737-014-0464-1.
17. Han, D.H.; Joe, K.H.; Na, C.; Lee, Y.S. Effect of genetic polymorphisms on smoking cessation: a trial of bupropion in Korean male smokers. *Psychiatr. Genet.* **2008**, *18*, 11–16, doi:10.1097/YPG.0B013E3282DF0939.

18. Higuchi, H.; Takahashi, H.; Kamata, M.; Yoshida, K. Influence of serotonergic/noradrenergic gene polymorphisms on nausea and sweating induced by milnacipran in the treatment of depression. *Neuropsychiatr. Dis. Treat.* **2009**, *5*, 393, doi:10.2147/NDT.S4369.
19. Hougardy, D.M.; Egberts, T.C.; van der Graaf, F.; Brenninkmeijer, V.J.; Derijks, L.J. Serotonin transporter polymorphism and bleeding time during SSRI therapy. *Br. J. Clin. Pharmacol.* **2008**, *65*, 761–766, doi:10.1111/J.13652125.2008.03098.X.
20. Hu, X.Z.; Rush, A.J.; Charney, D.; Wilson, A.F.; Sorant, A.J.; Papanicolaou, G.J.; Fava, M.; Trivedi, M.H.; Wisniewski, S.R.; Laje, G.; Paddock, S.; McMahon, F.J.; Manji, H.; Lipsky, R. H. Association between a functional serotonin transporter promoter polymorphism and citalopram treatment in adult outpatients with major depression. *Arch. Gen. Psychiatry* **2007**, *64*, 783–792, doi:10.1001/ARCHPSYC.64.7.783.
21. Huezo-Diaz, P.; Uher, R.; Smith, R.; Rietschel, M.; Henigsberg, N.; Marusic, A.; Mors, O.; Maier, W.; Hauser, J.; Souery, D.; Placentino, A.; Zobel, A.; Larsen, E.R.; Czerski, P.M.; Gupta, B.; Hoda, F.; Perroud, N.; Farmer, A.; Craig, I.; Aitchison, K. J.; McGuffin, P. Moderation of antidepressant response by the serotonin transporter gene. *Br. J. Psychiatry* **2009**, *195*, 30–38, doi:10.1192/BJP.BP.108.062521.
22. Ishiguro, S.; Watanabe, T.; Ueda, M.; Saeki, Y.; Hayashi, Y.; Akiyama, K.; Saito, A.; Kato, K.; Inoue, Y.; Shimoda, K. Determinants of pharmacodynamic trajectory of the therapeutic response to paroxetine in Japanese patients with panic disorder. *Eur. J. Clin. Pharmacol.* **2011**, *67*, 1213–1221, doi:10.1007/S00228-011-1073-9.
23. Joyce, P.R.; Porter, R.J.; Mulder, R.T.; Luty, S.E.; McKenzie, J.M.; Miller, A.L.; Kennedy, M.A. Reversed diurnal variation in depression: associations with a differential antidepressant response, tryptophan: large neutral amino acid ratio and serotonin transporter polymorphisms. *Psychol. Med.* **2005**, *35*, 511–517, doi:10.1017/S0033291704003861.
24. Kato, M.; Fukuda, T.; Wakeno, M.; Fukuda, K.; Okugawa, G.; Ikenaga, Y.; Yamashita, M.; Takekita, Y.; Nobuhara, K.; Azuma, J.; Kinoshita, T. Effects of the serotonin type 2A, 3A and 3B receptor and the serotonin transporter genes on paroxetine and fluvoxamine efficacy and adverse drug reactions in depressed Japanese patients. *Neuropsychobiology* **2006**, *53*, 186–195, doi:10.1159/000094727.
25. Kenna, G.A.; Zywiak, W.H.; McGeary, J.E.; Leggio, L.; McGeary, C.; Wang, S.; Grenga, A.; Swift, R.M. A withingroup design of nontreatment seeking 5-HTTLPR genotyped alcohol-dependent subjects receiving ondansetron and sertraline. *Alcohol. Clin. Exp. Res.* **2009**, *33*, 315–323, doi:10.1111/J.1530-0277.2008.00835.X.
26. Kenna, G.A.; Zywiak, W. H.; Swift, R.M.; McGeary, J.E.; Clifford, J.S.; Shoaff, J.R.; Fricchione, S.; Brickley, M.; Beaucage, K.; Haass-Koffler, C.L., Leggio, L. Ondansetron and sertraline may interact with 5-HTTLPR and DRD4 polymorphisms to reduce drinking in non-treatment seeking alcohol-dependent women: exploratory findings. *Alcohol* **2014**, *48*, 515–522, doi:10.1016/J.ALCOHOL.2014.04.005.
27. Kranzler, H.R.; Armeli, S.; Tennen, H.; Covault, J.; Feinn, R.; Arias, A.J.; Pettinati, H.; Oncken, C. A double-blind, randomized trial of sertraline for alcohol dependence: moderation by age of onset [corrected] and 5hydroxytryptamine transporter-linked promoter region genotype. *J. Clin. Psychopharmacol.* **2011**, *31*, 22–30, doi:10.1097/JCP.0B013E31820465FA.
28. Kranzler, H.R.; Armeli, S.; Tennen, H. Post-treatment outcomes in a double-blind, randomized trial of sertraline for alcohol dependence. *Alcohol. Clin. Exp. Res.* **2012**, *36*, 739–744, doi:10.1111/J.1530-0277.2011.01659.X.
29. Kranzler, H.R.; Armeli, S.; Tennen, H.; Covault, J. 5-HTTLPR genotype and daily negative mood moderate the effects of sertraline on drinking intensity. *Addict. Biol.* **2013**, *18*, 1024–1031, doi:10.1111/ADB.12007.
30. Kronenberg, S.; Apter, A.; Brent, D.; Schirman, S.; Melhem, N.; Pick, N.; Gothelf, D.; Carmel, M.; Frisch, A.; Weizman, A. Serotonin Transporter Polymorphism (5-HTTLPR) and Citalopram Effectiveness and Side Effects in Children with Depression and/or Anxiety Disorders. *J. Child Adolesc. Psychopharmacol.* **2007**, *17*, 741–750, doi:10.1089/cap.2006.0144.
31. Lanctôt, K.L.; Rapoport, M.J.; Chan, F.; Rajaram, R.D.; Strauss, J.; Sicard, T.; McCullagh, S.; Feinstein, A.; Kiss, A.; Kennedy, J.L.; Bassett, A.S.; Herrmann, N. Genetic predictors of response to treatment with citalopram in depression secondary to traumatic brain injury. *Brain Inj.* **2010**, *24*, 959–969, doi:10.3109/02699051003789229.
32. Lee, S.H.; Choi, T.K.; Lee, E.; Seok, J.H.; Lee, S.H.; Lee, H.S.; Kim, S.J. Serotonin transporter gene polymorphism associated with short-term treatment response to venlafaxine. *Neuropsychobiology* **2010**, *62*, 198–206, doi:10.1159/000319362.
33. Lee, E.J.; Oh, M.S.; Kim, J.S.; Chang, D.I.; Park, J.H.; Cha, J.K.; Heo, J.H.; Sohn, S.I.; Kim, D.E.; Kim, H.Y.; Kim, J.; Seo, W.K.; Lee, J.; Park, S.W.; Kim, Y.J.; Lee, B.C.; EMOTION investigators. Serotonin transporter gene

polymorphisms may be associated with poststroke neurological recovery after escitalopram use. *J. Neurol. Neurosurg. Psychiatry* **2018**, *89*, 271–276, doi:10.1136/JNNP-2017-316882.
34. Lenze, E.J.; Goate, A.M.; Nowotny, P.; Dixon, D.; Shi, P.; Bies, R.R.; Lotrich, F.K.; Rollman, B.L.; Shear, M.K.; Thompson, P.A.; Andreescu, C.; Pollock, B.G. Relation of serotonin transporter genetic variation to efficacy of escitalopram for generalized anxiety disorder in older adults. *J. Clin. Psychopharmacol.* **2010**, *30*, 672–677, doi:10.1097/JCP.0B013E3181FC2BEF.
35. Liu, Y.; Jia, L.; Jiang, S.M.; Chen, D.Y.; Song, J.S.; Xu, J. Serotonin Transporter Gene (SLC6A4) Polymorphism May Be Associated with Chinese Globus Pharyngeus and Its Antidepressant Effects. *Digestion* **2018**, *97*, 146–153, doi:10.1159/000484202.
36. Lohoff, F.W.; Narasimhan, S.; Rickels, K. Interaction between polymorphisms in serotonin transporter (SLC6A4) and serotonin receptor 2A (HTR2A) genes predict treatment response to venlafaxine XR in generalized anxiety disorder. *Pharmacogenomics J.* **2013**, *13*, 464–469, doi:10.1038/TPJ.2012.33.
37. Mandelli, L.; Mazza, M.; Martinotti, G.; Di Nicola, M.; Tavian, D.; Colombo, E.; Missaglia, S.; De Ronchi, D.; Colombo, R.; Janiri, L.; Serretti, A. Harm avoidance moderates the influence of serotonin transporter gene variants on treatment outcome in bipolar patients. *J. Affect. Disord.* **2009**, *119*, 205–209, doi:10.1016/J.JAD.2009.02.027.
38. Maron, E.; Tammiste, A.; Kallassalu, K.; Eller, T.; Vasar, V.; Nutt, D.J.; Metspalu, A. Serotonin transporter promoter region polymorphisms do not influence treatment response to escitalopram in patients with major depression. *Eur. Neuropsychopharmacol.* **2009**, *19*, 451–456, doi:10.1016/J.EURONEURO.2009.01.010.
39. Masoliver, E.; Menoyo, A.; Pérez, V.; Volpini, V.; Rio, E. D.; Pérez, J.; Alvarez, E.; Baiget, M. Serotonin transporter linked promoter (polymorphism) in the serotonin transporter gene may be associated with antidepressant-induced mania in bipolar disorder. *Psychiatr. Genet.* **2006**, *16*, 25–29, doi:10.1097/01.YPG.0000180684.26288.D7.
40. Miguita, K.; Cordeiro, Q.; Shavitt, R.G.; Miguel, E.C.; Vallada, H. Association study between genetic monoaminergic polymorphisms and OCD response to clomipramine treatment. *Arq. Neuropsiquiatr.* **2011**, *69*, 283–287, doi:10.1590/S0004-282X2011000300003.
41. Monteleone, P.; Santonastaso, P.; Tortorella, A.; Favaro, A.; Fabrazzo, M.; Castaldo, E.; Caregaro, L.; Fuschino, A.; Maj, M. Serotonin transporter polymorphism and potential response to SSRIs in bulimia nervosa. *Mol. Psychiatry* **2005**, *10*, 716–718, doi:10.1038/SJ.MP.4001683.
42. Murata, Y.; Kobayashi, D.; Imuta, N.; Haraguchi, K.; Ieiri, I.; Nishimura, R.; Koyama, S.; Mine, K. Effects of the serotonin 1A, 2A, 2C, 3A, and 3B and serotonin transporter gene polymorphisms on the occurrence of paroxetine discontinuation syndrome. *J. Clin. Psychopharmacol.* **2010**, *30*, 11–17, doi:10.1097/JCP.0B013E3181C8AE80.
43. Murphy, G.M.; Jr, Hollander, S.B.; Rodrigues, H.E.; Kremer, C.; Schatzberg, A. F. Effects of the serotonin transporter gene promoter polymorphism on mirtazapine and paroxetine efficacy and adverse events in geriatric major depression. *Arch. Gen. Psychiatry* **2004**, *61*, 1163–1169, doi:10.1001/ARCHPSYC.61.11.1163.
44. Mushtaq, D.; Ali, A.; Margoob, M.A.; Murtaza, I.; Andrade, C. Association between serotonin transporter gene promoter-region polymorphism and 4- and 12-week treatment response to sertraline in posttraumatic stress disorder. *J. Affect. Disord.* **2012**, *136*, 955–962, doi:10.1016/J.JAD.2011.08.033.
45. Najjar, F.; Owley, T.; Mosconi, M.W.; Jacob, S.; Hur, K.; Guter, S.J.; Sweeney, J.A.; Gibbons, R.D.; Cook, E.H.; Bishop, J. R. Pharmacogenetic Study of Serotonin Transporter and 5HT2A Genotypes in Autism. *J. Child Adolesc. Psychopharmacol.* **2015**, *25*, 467–474, doi:10.1089/CAP.2014.0158.
46. Ng, C.; Sarris, J.; Singh, A.; Bousman, C.; Byron, K.; Peh, L.H.; Smith, D.J.; Tan, C.H.; Schweitzer, I. Serotonin transporter polymorphisms and clinical response to sertraline across ethnicities. *Prog. Neuropsychopharmacol. Biol. Psychiatry* **2006**, *30*, 953–957, doi:10.1016/J.PNPBP.2006.02.015.
47. Ng, C.; Sarris, J.; Singh, A.; Bousman, C.; Byron, K.; Peh, L.H.; Smith, D.J.; Tan, C.H.; Schweitzer, I. Pharmacogenetic polymorphisms and response to escitalopram and venlafaxine over 8 weeks in major depression. *Hum. Psychopharmacol.* **2013**, *28*, 516–522, doi:10.1002/HUP.2340.
48. Ng, C.H.; Bousman, C.; Smith, D.J.; Dowling, N.; Byron, K.; King, J.; Sarris, J. A Prospective Study of Serotonin and Norepinephrine Transporter Genes and the Response to Desvenlafaxine Over 8 Weeks in Major Depressive Disorder. *Pharmacopsychiatry* **2016**, *49*, 210–212, doi:10.1055/S-0042-103968.
49. Owley, T.; Brune, C.W.; Salt, J.; Walton, L.; Guter, S.; Ayuyao, N.; Gibbons, R.D.; Leventhal, B.L.; Cook, E.H. A pharmacogenetic study of escitalopram in autism spectrum disorders. *Autism Res.* **2010**, *3*, 1–7, doi:10.1002/AUR.109.

50. Ozbek, E.; Otunctemur, A.; Simsek, A.; Polat, E.C.; Ozcan, L.; Köse, O.; Cekmen, M. Genetic polymorphism in the serotonin transporter gene-linked polymorphic region and response to serotonin reuptake inhibitors in patients with premature ejaculation. *Clinics (Sao Paulo).* **2014**, *69*, 710–713, doi:10.6061/CLINICS/2014(11)01.
51. Park, H.J.; Kim, S.K.; Kang, W.S.; Kim, Y.J.; Cho, A.R.; Park, J.K. Potential involvement of NET polymorphism in serotonin/norepinephrine reuptake inhibitor response in panic disorder. *Nord. J. Psychiatry* **2016**, *70*, 314–317, doi:10.3109/08039488.2015.1089321.
52. Perlis, R.H.; Mischoulon, D.; Smoller, J.W.; Wan, Y.J.; Lamon-Fava, S.; Lin, K.M.; Rosenbaum, J.F.; Fava, M. Serotonin transporter polymorphisms and adverse effects with fluoxetine treatment. *Biol. Psychiatry* **2003**, *54*, 879–883, doi:10.1016/S0006-3223(03)00424-4.
53. Perna, G.; Favaron, E.; Di Bella, D.; Bussi, R.; Bellodi, L. Antipanic efficacy of paroxetine and polymorphism within the promoter of the serotonin transporter gene. *Neuropsychopharmacology* **2005**, *30*, 2230–2235, doi:10.1038/SJ.NPP.1300822.
54. Peters, M.E.; Vaidya, V.; Drye, L.T.; Devanand, D.P.; Mintzer, J.E.; Pollock, B.G.; Porsteinsson, A.P.; Rosenberg, P.B.; Schneider, L.S.; Shade, D.M.; Weintraub, D.; Yesavage, J.; Lyketsos, C.G.; Avramopoulos, D.; CitAD Research Group. Citalopram for the Treatment of Agitation in Alzheimer Dementia: Genetic Influences. *J. Geriatr. Psychiatry Neurol.* **2016**, *29*, 59–64, doi:10.1177/0891988715601735.
55. Popp, J.; Leucht, S.; Heres, S.; Steimer, W. Serotonin transporter polymorphisms and side effects in antidepressant therapy--a pilot study. *Pharmacogenomics* **2006**, *7*, 159–166, doi:10.2217/14622416.7.2.159.
56. Putzhammer, A.; Schoeler, A.; Rohrmeier, T.; Sand, P.; Hajak, G.; Eichhammer, P. Evidence of a role for the 5HTTLPR genotype in the modulation of motor response to antidepressant treatment. *Psychopharmacology (Berl).* **2005**, *178*, 303–308, doi:10.1007/S00213-004-1995-3.
57. Quaak, M.; van Schayck, C.P.; Postma, D.S.; Wagena, E.J.; van Schooten, F. J. Genetic variants in the serotonin transporter influence the efficacy of bupropion and nortriptyline in smoking cessation. *Addiction* **2012**, *107*, 178–187, doi:10.1111/J.1360-0443.2011.03534.X.
58. Rahikainen, A.L.; Majaharju, S.; Haukka, J.; Palo, J. U.; Sajantila, A. Serotonergic 5HTTLPR/rs25531 s-allele homozygosity associates with violent suicides in male citalopram users. *Am. J. Med. Genet. B. Neuropsychiatr. Genet.* **2017**, *174*, 691–700, doi:10.1002/AJMG.B.32553.
59. Rotberg, B.; Kronenberg, S.; Carmel, M.; Frisch, A.; Brent, D.; Zalsman, G.; Apter, A.; Weizman, A. Additive effects of 5-HTTLPR (serotonin transporter) and tryptophan hydroxylase 2 G-703T gene polymorphisms on the clinical response to citalopram among children and adolescents with depression and anxiety disorders. *J. Child Adolesc. Psychopharmacol.* **2013**, *23*, 117–122, doi:10.1089/CAP.2012.0020.
60. Rousseva, A.; Henry, C.; van den Bulke, D.; Fournier, G.; Laplanche, J. L.; Leboyer, M.; Bellivier, F.; Aubry, J.M.; Baud, P.; Boucherie, M.; Buresi, C.; Ferrero, F.; Malafosse, A. Antidepressant-induced mania, rapid cycling and the serotonin transporter gene polymorphism. *Pharmacogenomics J.* **2003**, *3*, 101–104, doi:10.1038/SJ.TPJ.6500156.
61. Saeki, Y.; Watanabe, T.; Ueda, M.; Saito, A.; Akiyama, K.; Inoue, Y.; Hirokane, G.; Morita, S.; Yamada, N.; Shimoda, K. Genetic and pharmacokinetic factors affecting the initial pharmacotherapeutic effect of paroxetine in Japanese patients with panic disorder. *Eur. J. Clin. Pharmacol.* **2009**, *65*, 685–691, doi:10.1007/S00228-009-0633-8.
62. Sahraian, S.; Babashams, M.; Reza-Soltani, P.; Najmabadi, H.; Kahrizi, K.; Gorgani, S.H. Serotonin Transporter Polymorphism (5-HTTLPR) and Citalopram Effectiveness in Iranian Patients with Major Depressive Disorder. *Iran. J. Psychiatry* **2013**, *8*, 86.
63. Saiz-Rodríguez, M.; Belmonte, C.; Román, M.; Ochoa, D.; Koller, D.; Talegón, M.; Ovejero-Benito, M.C.; LópezRodríguez, R.; Cabaleiro, T.; Abad-Santos, F. Effect of Polymorphisms on the Pharmacokinetics, Pharmacodynamics and Safety of Sertraline in Healthy Volunteers. *Basic Clin. Pharmacol. Toxicol.* **2018**, *122*, 501–511, doi:10.1111/BCPT.12938.
64. Salem, A.M.; Kamel, I.I.; Rashed, L.A.; GamalEl Din, S.F. Effects of paroxetine on intravaginal ejaculatory latency time in Egyptian patients with lifelong premature ejaculation as a function of serotonin transporter polymorphism. *Int. J. Impot. Res.* **2017**, *29*, 7–11, doi:10.1038/IJIR.2016.36.
65. Secher, A.; Bukh, J.; Bock, C.; Koefoed, P.; Rasmussen, H.B.; Werge, T.; Kessing, L.V.; Mellerup, E. Antidepressivedrug-induced bodyweight gain is associated with polymorphisms in genes coding for COMT and TPH1. *Int. Clin. Psychopharmacol.* **2009**, *24*, 199–203, doi:10.1097/YIC.0B013E32832D6BE2.
66. Serretti, A.; Zanardi, R.; Rossini, D.; Cusin, C.; Lilli, R.; Smeraldi, E. Influence of tryptophan hydroxylase and serotonin transporter genes on fluvoxamine antidepressant activity. *Mol. Psychiatry* **2001**, *6*, 586–592, doi:10.1038/SJ.MP.4000876.

67. Serretti, A.; Cusin, C.; Rossini, D.; Artioli, P.; Dotoli, D.; Zanardi, R. Further evidence of a combined effect of SERTPR and TPH on SSRIs response in mood disorders. *Am. J. Med. Genet. B. Neuropsychiatr. Genet.* **2004**, *129B*, 36– 40, doi:10.1002/AJMG.B.30027.
68. Serretti, A.; Mandelli, L.; Lorenzi, C.; Pirovano, A.; Olgiati, P.; Colombo, C.; Smeraldi, E. Serotonin transporter gene influences the time course of improvement of "core" depressive and somatic anxiety symptoms during treatment with SSRIs for recurrent mood disorders. *Psychiatry Res.* **2007**, *149*, 185–193, doi:10.1016/J.PSYCHRES.2006.03.020.
69. Silva, H.; Iturra, P.; Solari, A.; Villarroel, J.; Jerez, S.; Jiménez, M.; Galleguillos, F.; Bustamante, M. L. Fluoxetine response in impulsive-aggressive behavior and serotonin transporter polymorphism in personality disorder. *Psychiatr. Genet.* **2010**, *20*, 25–30, doi:10.1097/YPG.0B013E328335125D.
70. Smits, K.; Smits, L.; Peeters, F.; Schouten, J.; Janssen, R.; Smeets, H.; van Os, J.; Prins, M. Serotonin transporter polymorphisms and the occurrence of adverse events during treatment with selective serotonin reuptake inhibitors. *Int. Clin. Psychopharmacol.* **2007**, *22*, 137–143, doi:10.1097/YIC.0B013E328014822A.
71. Staeker, J.; Leucht, S.; Laika, B.; Steimer, W. Polymorphisms in serotonergic pathways influence the outcome of antidepressant therapy in psychiatric inpatients. *Genet. Test. Mol. Biomarkers* **2014**, *18*, 20–31, doi:10.1089/GTMB.2013.0217.
72. Stein, M.B.; Seedat, S.; Gelernter, J. Serotonin transporter gene promoter polymorphism predicts SSRI response in generalized social anxiety disorder. *Psychopharmacology (Berl).* **2006**, *187*, 68–72, doi:10.1007/S00213-006-0349-8.
73. Stein, M. B.; Keshaviah, A.; Haddad, S. A.; Van Ameringen, M.; Simon, N.M.; Pollack, M.H.; Smoller, J. W. Influence of RGS2 on sertraline treatment for social anxiety disorder. *Neuropsychopharmacology* **2014**, *39*, 1340–1346, doi:10.1038/NPP.2013.301.
74. Strohmaier, J.; Wüst, S.; Uher, R.; Henigsberg, N.; Mors, O.; Hauser, J.; Souery, D.; Zobel, A.; Dernovsek, M.Z.; Streit, F.; Schmäl, C.; Kozel, D.; Placentino, A.; Farmer, A.; McGuffin, P.; Aitchison, K.J.; Rietschel, M. Sexual dysfunction during treatment with serotonergic and noradrenergic antidepressants: clinical description and the role of the 5-HTTLPR. *World J. Biol. Psychiatry* **2011**, *12*, 528–538, doi:10.3109/15622975.2011.559270.
75. Sugie, Y.; Sugie, H.; Fukuda, T.; Ito, M.; Sasada, Y.; Nakabayashi, M.; Fukashiro, K.; Ohzeki, T. Clinical efficacy of fluvoxamine and functional polymorphism in a serotonin transporter gene on childhood autism. *J. Autism Dev. Disord.* **2005**, *35*, 377–85.
76. Takahashi, H.; Yoshida, K.; Ito, K.; Sato, K.; Kamata, M.; Higuchi, H.; Shimizu, T.; Ito, K.; Inoue, K.; Tezuka, T.; Suzuki, T.; Ohkubo, T.; Sugawara, K. No association between the serotonergic polymorphisms and incidence of nausea induced by fluvoxamine treatment. *Eur. Neuropsychopharmacol.* **2002**, *12*, 477–481, doi:10.1016/S0924977X(02)00056-1.
77. Tomita, T.; Yasui-Furukori, N.; Nakagami, T.; Tsuchimine, S.; Ishioka, M.; Kaneda, A.; Sugawara, N.; Kaneko, S. The influence of 5-HTTLPR genotype on the association between the plasma concentration and therapeutic effect of paroxetine in patients with major depressive disorder. *PLoS One* **2014**, *9*, doi:10.1371/JOURNAL.PONE.0098099.
78. Wilkie, M.J.; Smith, G.; Day, R.K.; Matthews, K.; Smith, D.; Blackwood, D.; Reid, I.C.; Wolf, C. R. Polymorphisms in the SLC6A4 and HTR2A genes influence treatment outcome following antidepressant therapy. *Pharmacogenomics J.* **2009**, *9*, 61–70, doi:10.1038/SJ.TPJ.6500491.
79. Won, E.S.; Chang, H.S.; Lee, H.Y.; Ham, B.J.; Lee, M.S. Association between serotonin transporter-linked polymorphic region and escitalopram antidepressant treatment response in Korean patients with major depressive disorder. *Neuropsychobiology* **2012**, *66*, 221–229, doi:10.1159/000341876.
80. Wong, W.M.; Hasemann, S.; Schwarz, M.; Zill, P.; Koller, G.; Soyka, M.; Preuss, U.W. Citalopram neuropharmacological challenge in alcohol-dependent patients and controls: pharmacogenetic, endocrine and psychobehavioral results. *Pharmacopsychiatry* **2008**, *41*, 72–78, doi:10.1055/S-2007-1004595.
81. Yevtushenko, O.O.; Oros, M.M.; Reynolds, G.P. Early response to selective serotonin reuptake inhibitors in panic disorder is associated with a functional 5-HT1A receptor gene polymorphism. *J. Affect. Disord.* **2010**, *123*, 308–311, doi:10.1016/J.JAD.2009.09.007.
82. Zanardi, R.; Serretti, A.; Rossini, D.; Franchini, L.; Cusin, C.; Lattuada, E.; Dotoli, D.; Smeraldi, E. Factors affecting fluvoxamine antidepressant activity: influence of pindolol and 5-HTTLPR in delusional and nondelusional depression. *Biol. Psychiatry* **2001**, *50*, 323–330, doi:10.1016/S0006-3223(01)01118-0.
